# Hybrid bagging and boosting with SHAP based feature selection for enhanced predictive modeling in intrusion detection systems

**DOI:** 10.1038/s41598-024-81151-1

**Published:** 2024-12-17

**Authors:** Usman Ahmed, Zheng Jiangbin, Ahmad Almogren, Muhammad Sadiq, Ateeq Ur Rehman, M. T. Sadiq, Jaeyoung Choi

**Affiliations:** 1 School of Software, Northwestern Ploytechnical University, Xian, 710072 China; 2 Chair of Cyber Security, Department of Computer Science, College of Computer and Information Sciences, King Saud University, Riyadh, 11633 Saudi Arabia; 3 School of Computer Science and Electronic Engineering, University of Essex, Colchester Campus, United Kingdom; 4 School of Computing, Gachon University, Seongnam-si, 13120 Republic of Korea; 5 Applied Science Research Center, Applied Science Private University, Amman, Jordan; 6 Computer Science and Engineering, Saveetha School of Engineering, Saveetha Institute of Medical and Technical Sciences, Chennai, India; 7 University Center for Research and Development, Chandigarh University, Mohali, India

**Keywords:** Bagging and Boosting, Network Security, Intrusion Detection and Prevention Systems, Explainable AI, SHAP Feature Selection, Engineering, Computer science

## Abstract

The novelty and growing sophistication of cyber threats mean that high accuracy and interpretable machine learning models are needed more than ever before for Intrusion Detection and Prevention Systems. This study aims to solve this challenge by applying Explainable AI techniques, including Shapley Additive explanations feature selection, to improve model performance, robustness, and transparency. The method systematically employs different classifiers and proposes a new hybrid method called Hybrid Bagging-Boosting and Boosting on Residuals. Then, performance is taken in four steps: the multistep evaluation of hybrid ensemble learning methods for binary classification and fine-tuning of performance; feature selection using Shapley Additive explanations values retraining the hybrid model for better performance and reducing overfitting; the generalization of the proposed model for multiclass classification; and the evaluation using standard information metrics such as accuracy, precision, recall, and F1-score. Key results indicate that the proposed methods outperform state-of-the-art algorithms, achieving a peak accuracy of 98.47% and an F1 score of 96.19%. These improvements stem from advanced feature selection and resampling techniques, enhancing model accuracy and balancing precision and recall. Integrating Shapley Additive explanations-based feature selection with hybrid ensemble methods significantly boosts the predictive and explanatory power of Intrusion Detection and Prevention Systems, addressing common pitfalls in traditional cybersecurity models. This study paves the way for further research on statistical innovations to enhance Intrusion Detection and Prevention Systems performance.

## Introduction

The level of complexity and sophistication within cyber threats has surged by the day; hence, advanced intrusion detection and prevention systems were required to identify and mitigate security breaches^1^. The common Intrusion Detection and Prevention Systems (IDPS), heavily dependent on signature-based and anomaly-based detection techniques, are often outpaced by the dynamic character of contemporary attacks^2^. To address these challenges, machine learning and deep learning have been combined into Intrusion Detection and Prevention Systems frameworks, offering improved detection through education and collaboration to deal with new threats^3^.

Explainable Artificial Intelligence (XAI) is a crucial research area focusing on the inner workings of black-box machine learning and deep learning models^4^. The Explainable AI approach makes those models more interpretable and explainable to people while decreasing model performance^5^. Regarding intrusion detection and prevention, Explainable AI helps security professionals understand why a particular decision was made, which helps verify the model behavior, identifies potential biases, and assures its regulatory compliance^6^.

One of the popular methods is the use of feature-important techniques, with Shapley Additive explanations (SHAP)^7^ being one of the popular methods for quantitatively evaluating the contribution of each feature to model predictions^8^. It has been incredibly effective at indicating the portions of network traffic data that point to possible intrusions, making security analysts more concerned with the most crucial parts of data. Furthermore, the integration of SHAP over some of the more well-acknowledged machine learning classifiers, such as Decision Tree (DT), Logistic Regression (LR), Random Forest (RF), Stochastic Gradient Descent (SGD), Light Gradient Boosting Machines (LGBM), Extreme Gradient Boosting (XGB) and AdaBoost (ADB)^9,10^, gives a general framework for evaluating and enhancing the performance and interpretability of IDPS.

Although the development of ensemble learning models and feature selection is phenomenal, robust performance and interpretability in classification remain challenging. Traditional algorithms often aim at accuracy with very little concern for transparency. Hence, understanding model behavior and the importance of features is cumbersome^11^. Besides, the existing methods may not avoid overfitting issues or explore the most informative features while migrating from binary to multiclass classification problems. The existing models also do not propose a stepwise approach in a structured manner, which can integrate feature selection with ensemble techniques to lead to better performance and more insights into the black box^12^.

Thus, a method that can address the limitations mentioned by incorporating structured evaluation of ensemble models combined with informed feature selection based on SHAP values and hierarchical classification strategy is required. This research, therefore, attempts to fill this gap by proposing a multi-fold method that enhances model robustness and gives clear insights into feature importance for this innovative approach.

This paper presents Explainable AI techniques to enhance the interpretability and performance of machine learning models for IDPS. This study uses the SHAP feature selection method concerning the impact on model accuracy, robustness, and interpretability. Following an established approach where best practices are pointed out and common pitfalls when organizations try to incorporate Explainable AI into existing cybersecurity frameworks. The study systematically compares the method across a range of classifiers. It provides comprehensive insights into how XAI can be used to build more transparent and trustworthy IDPS.

### Challenges and limitations

The proposed method has the following limitations:

- Computational Complexity: The interpretability of Ensemble models with SHAP and LIME requires great computational resources; thus, it impairs scalability and real-time applicability in resource-constrained environments.
- Generalization issues: The model will entirely depend on the CIC-IDS2017 dataset, making it fail to generalize on various network environments or recent attack types that are not included.
- Error Propagation in Hierarchical Classification: The two-step process could lead to error propagation. Misclassifications present on the binary level can negatively influence the accuracy of the multiclass classifications.
- Instability of SHAP Values: Feature importance computed using SHAP values is sensitive to model parameters and data distribution. Therefore, feature rankings across different scenarios may result in inconsistent features.
- Interpretability Challenges: While SHAP and LIME improve explainability, they are likely to yield imprecise explanations from hard-to-understand ensemble model processes in which meaningful insight is hard to extract.
- Overfitting Risk: Even so, several ensemble methods are prone to overfitting or modeling noise from training data instead of generalization to unseen data, even with feature selection in place to prevent such scenarios.
- High Computational Demand: The computations of the algorithm lead to unacceptable latencies for real-time detection on resource-poor IDS hardware.
- Challenges on Real-time Adaptation: Adapting the batch-designed algorithm for data streaming and attack updates at a high frequency is challenging.
- Complexity of the Integration: The integration in existing IDS frameworks will be complicated and might cause instability.
- Performance trade-offs: In real-time interpretability, computational load adds up, so there’s always some performance-transparency trade-off in speed; error propagation can reduce accuracy.

Overcoming these limitations would involve further work on computational efficiency, testing on various datasets, which would enhance the generalisability of the same, refinement in the classification strategy so that error propagation is reduced, stability in feature importance rankings, and real-world deployment tests.

### Contribution

The main contribution is as follows:

- Multi-fold method: This paper recommends a stepwise systematic approach to evaluating an array of ensemble learning models for robust performance and interpretability.
- Selection of features based on SHAP values: SHAP values help identify the informative features, so models were trained using relevant information that will make a difference in performance and save against overfitting.
- Two-step initial binary classification: The first classification is the initial binary classification process to fine-tune further and get an idea about model behavior.
- Stepwise extended multiclass classification: This is a hierarchical approach of the two-class classification type to bring out the generality of model performance.
- Approaches:Two approaches have been used: Hybrid Bagging-Boosting and Boosting on Residuals.
- Evaluation:The models will be further evaluated against evaluation metrics of interest, such as accuracy, precision, recall, and F1 score. At the same time, SHAP values will emphasize features’ interpretability.
- Explanation: The importance of the SHAP and LIME values feature explains the models’ behaviour, which is viewable in the surfacing feature to authorize transparency and validation of the model predictions.

### Organization

The rest of this paper is organized as follows: Related work is summarized in section "Related work". Section "Methods" discusses the Proposed Methodology, Bagging, and Boosting, Boosting on Residuals, Datasets, Data Processing, and Implementation details. Results, Confusion Matrix, Explainable AI Analysis, Discussion, Performance analysis, and Comparative study are discussed in section "Results and discussion". Section "Conclusion and future direction" concludes the study with future work.

## Related work

Machine-augmented IDSs have been discussed in the literature and have tremendously improved the bar of detection and prevention in the cyber-threat landscape. Earlier studies predominately aimed at designing classifiers using supervised learning techniques for classifying network traffic into benign or malicious classes with the help of labeled datasets. For instance, in conventional machine learning, simple and interpretable properties were DTs^13^ and LR^14^. However, they could not handle the high dimensionality and complexity of network traffic data, soon providing the impetus for discovering better-performing and more robust methods, such as RFs^15^ and GBMs^16^.

The most recent development is applying DL^17^ techniques to IDS. In particular, CNN^18^ and RNN^19^ have been used for end-to-end feature learning from raw network traffic data in the open environment, improving attack accuracy. For instance,^20^ allowed LSTM^21^ networks to learn the temporal relationships in network attack data for anomaly detection. However, these DL models’ success equals their failing point, as their mysterious black-box characteristics propagate the trust problem.

Many studies have further demonstrated the utility of bagging and boosting, especially for IDPSs. For instance, improved intrusion detection using a bagging technique to improve accuracy and reduce FPR was demonstrated by Khoshgoftaar^22^. Another study using boosting methods such as AdaBoost^23^ and GB^16^ for intrusion detection showed that boosting algorithms can overcome the issue of robustness by finding non-linear classification models with high accuracy and generalization ability. In addition, the GB technique can detect patterns of an advanced attack, often not detected by a single classifier. Hybrid ensembles that combine bagging and boosting techniques had even better performance^24^.

Reference^25^ investigate how optimized machine learning models with modified Firefly algorithm can be integrated to enhance the security of IoT-based healthcare systems. The authors use SHAP to interpret which features influence intrusion detection for optimizing performance. Thus, they want to contribute to solving questions about specific challenges related to sustainability in Healthcare 4.0. It was observed with SHAP analysis that significant improvements in the detection of security issues are substantial, making this a beneficial work to understand how SHAP is used in intrusion detection frameworks, particularly those applied to healthcare.

Reference^26^ proposed an advanced intrusion detection technique in cyber-physical systems (CPSs) with the help of meta-heuristics incorporated by the Quantum Dwarf Mongoose Optimization (QDMO) algorithm and ensemble deep learning models. These include Convolution Residual Networks, Deep Belief Networks, and Deep Autoencoders. This was to improve intrusion detection accuracy. This work focuses on effective feature selection to depict how QDMO can scale up attack detection with reduced false positives in the CPS environment.

The paper^27^ proposes a new paradigm in intrusion detection in IoT networks by using a hybrid CNN with the XGBoost model optimized via the Modified Reptile Search Algorithm. In this regard, it aims to maximize intrusion detection performance by identifying relevant scalable features and optimizing model parameters. This implies that feature selection enhanced with RSA increased intrusion detection accuracy while SHAP analysis provides interpretability in decision-making.

Reference^28^ presents a two-step pipeline for intrusion detection in industrial cyber-physical systems, embedding explainable AI with SHAP for enhanced interpretability. The model leverages an improved Krill Herd Optimization technique within the feature selection process to improve the classification of CPS environment attacks. Including SHAP will help cybersecurity experts understand the model’s decisions and provide trustworthy explanations that can help build trust in the system’s performance.

Reference^29^ propose a hybrid AI framework for IoT security that integrates the sine-cosine metaheuristically tuned CNN-ELM model. This approach has been oriented toward feature selection to enhance intrusion detection. Also, SHAP analysis is included to obtain explanations from the model decisions, where the security expert could underline the most influential factors on a given classification. Results showed this concept outperformed the traditional idea in threat detection and mitigation of securities using IoT.

Compared to related studies, the proposed method stands out for its systematic, stepwise use of SHAP for feature selection, interpretability, and validation, coupled with multiclass classification for broader model generality. While other works also employ SHAP (e.g., Refs.^25,29^) or ensemble models (e.g.,Refs.^26,27^), they primarily focus on metaheuristic optimization rather than emphasis on transparency and robust classification. The proposed approach uniquely integrates hybrid bagging-boosting with a clear focus on feature importance and model explanation, setting it apart from optimization-driven methods.

## Materials

The proposed research uses open-source dataset, which is presented in the next section.

### Dataset 1

This research uses the CIC-IDS2017 dataset to develop and evaluate the intrusion detection models^30^. Developed by the Canadian Institute for Cybersecurity, CIC-IDS2017 is a complete network traffic dataset comprising both normal behavior and attack scenarios. It captured actual traffic that genuinely reflected user behavior and network conditions. It ranges over the different attack categories, including DoS and DDoS, brute force on SSH and FTP, Heartbleed exploits, botnet activities, and web attacks like SQL injection and cross-site scripting XSS. Every example of data is labeled as normal or some specific type of attack, facilitating both binary and multiclass classification tasks^31^.

The key benefit of the CIC-IDS2017 dataset is its feature richness. It offers 80 network flow features extracted using the tool CIC-FlowMeter, which covers features about the flow duration, total bytes, packet counts, and several statistical measures. Such a rich set of features is significant for in-depth analysis and model training, providing a clear insight into applying SHAP values in model predictions and effective feature selection.

Selecting the CIC-IDS2017 dataset is deliberate and strategic. Firstly, it is a relatively modern dataset with contemporary vectors in modern cybersecurity. This means the proposed model will be trained on data depicting current threats and not rely on older datasets such as KDD’99 or NSL-KDD, which no longer contain prevalent attacks. These datasets also face severe problems of redundancy and imbalance issues. The CIC-IDS2017 dataset is the critical choice because it offers comprehensive attack coverage; therefore, the models developed can generalize well in different intrusion scenarios and be practically applied.

Additionally, the dataset represents user behavior and network interactions, bridging the gap between experimental results and real-world performance. Its rich feature setting is especially suited for SHAP value application; therefore, model predictions and feature selection can be effectively made. Moreover, meaningful comparisons with other studies and benchmarks could be assured because it is one of the benchmark datasets in the particular area.

Other datasets, such as UNSW-NB15, provide representations for both normal and malicious traffic; however, they lack certain types of modern attacks and do not have a wide variety of specific attack classes. The other datasets, like the DARPA sets, were generated in controlled environments, which cannot reflect the current network complexities and lack some modern attack methods. Therefore, selecting the CIC-IDS2017 dataset ensures that the research is relevant and based on comprehensive data to solve the limitations found thus far in all other datasets. Consequently, it enhances the validity of results and the applicability of the models in the real world of intrusion detection. Table 1 shows the concise summary of the CIC-IDS2017 Wednesday attack subset.

**Table 1:** Concise Summary of Wednesday Attack.

Details of df8	Distribution of Target Variable in df8
Number of Rows: 692,703	BENIGN: 440,031
Number of Columns: 79	DoS Hulk: 231,073
Data Types: Float64 (24 columns),	DoS GoldenEye: 10,293
Int64 (54 columns),	DoS Slowloris: 5,796
Object (1 column)	DoS Slowhttptest: 5,499
Memory Usage: 417.5+ MB	Heartbleed: 11

### Handling Imbalanced dataset

Handling such an imbalanced dataset creates an essential task in intrusion detection systems. CIC-IDS2017 will contain some attack classes with a far lower occurrence rate than others. The presence of such classes is challenging for any model because it is highly biased toward the majority class. This may cause a considerable reduction in the performance of detecting these rare attacks. Handling this requires addressing the issue at the pre-processing stage and adaptation in the models themselves.

The most effective methodology for dealing with class imbalance involves resampling. Oversampling techniques, such as SMOTE and ADASYN^32^, interpolate between existing samples to create synthetic samples of minority classes. In this way, it represents the minority class more significantly in a dataset. It ensures that the model gets adequate training data on each class, making it less prone to bias related to the majority class. Undersampling involves the reduction of majority class samples by randomly removing them, in which case one gets a balanced training dataset, likely at the cost of losing some valuable information. Hybrid sampling combines both to maintain a balance while preserving essential data characteristics.

In addition to remedies at the data level, there are strategies at the algorithm level that might improve model performance when using imbalanced datasets. The first is adjusting class weights during model training by assigning more weight to minority classes so that the misclassification of those classes carries a higher penalty^33^. This approach is beneficial in such algorithms as RFs, SVMs, and XGBoost because the adjustment of weights is straightforward. Other potent techniques are ensemble methods specific to imbalanced datasets, such as Balanced Random Forests or EasyEnsemble. These methods give much more significant focus to the under-represented classes by creating many random subsets weighted toward the minority classes to help improve the generalization of a model^34^.

Class-specific performance metrics are critical in evaluating a model’s performance on imbalanced data. Precision and Recall give insight into the model performance on the minority classes; at the same time, AUC-ROC is about the overall classification capability across different thresholds, thus making it more comprehensive for imbalanced cases.

To address the challenge of handling imbalanced datasets, visualizations such as t-SNE projections and pair plots provide critical insights into the distribution of instances across classes in the CIC-IDS2017 dataset.

**Fig. 1:**
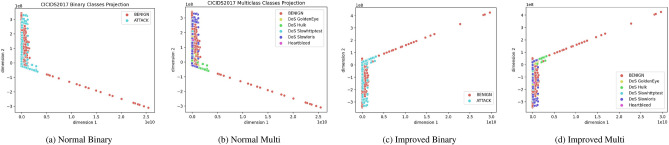
CIC-IDS2017 Class Projections.

**Fig. 2:**
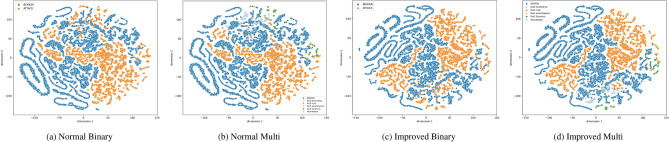
Four images in a single row.

Figures 1a,b and 2a,b t-SNE projection plots of binary and multiclass settings visually represent class distribution and separability in high-dimensional space. The imbalance in the dataset is apparent, especially in the binary classification where the “BENIGN” class has significantly more points than the “ATTACK” class. Similarly, in the multiclass projections, certain attack types (such as DoS Hulk) appear in greater concentrations than others (like Heartbleed), indicating the imbalance across various attack classes. This visualization highlights how imbalance can lead to overlapping clusters, making classification challenging, especially without data handling techniques like resampling. Initially, there is more overlap between benign and attack traffic, and then the enhanced clusters become more separated, reflecting higher accuracy in binary and multiclass classification.

Figures 1c,d and 2c,d visualizations reflect improvements after addressing class imbalance using SMOTE. In the binary classification, BENIGN and ATTACK classes are more clearly separated, with well-defined clusters, indicating that SMOTE has balanced the data and enhanced the model’s ability to distinguish between benign and malicious traffic. In the multiclass setting, different attack types, such as DoS GoldenEye and Heartbleed, are more distinct, with tighter and more separated clusters, suggesting that SMOTE helped mitigate the skew in attack type distributions, allowing the model to better differentiate between the various classes and improve overall classification performance.

**Fig. 3:**
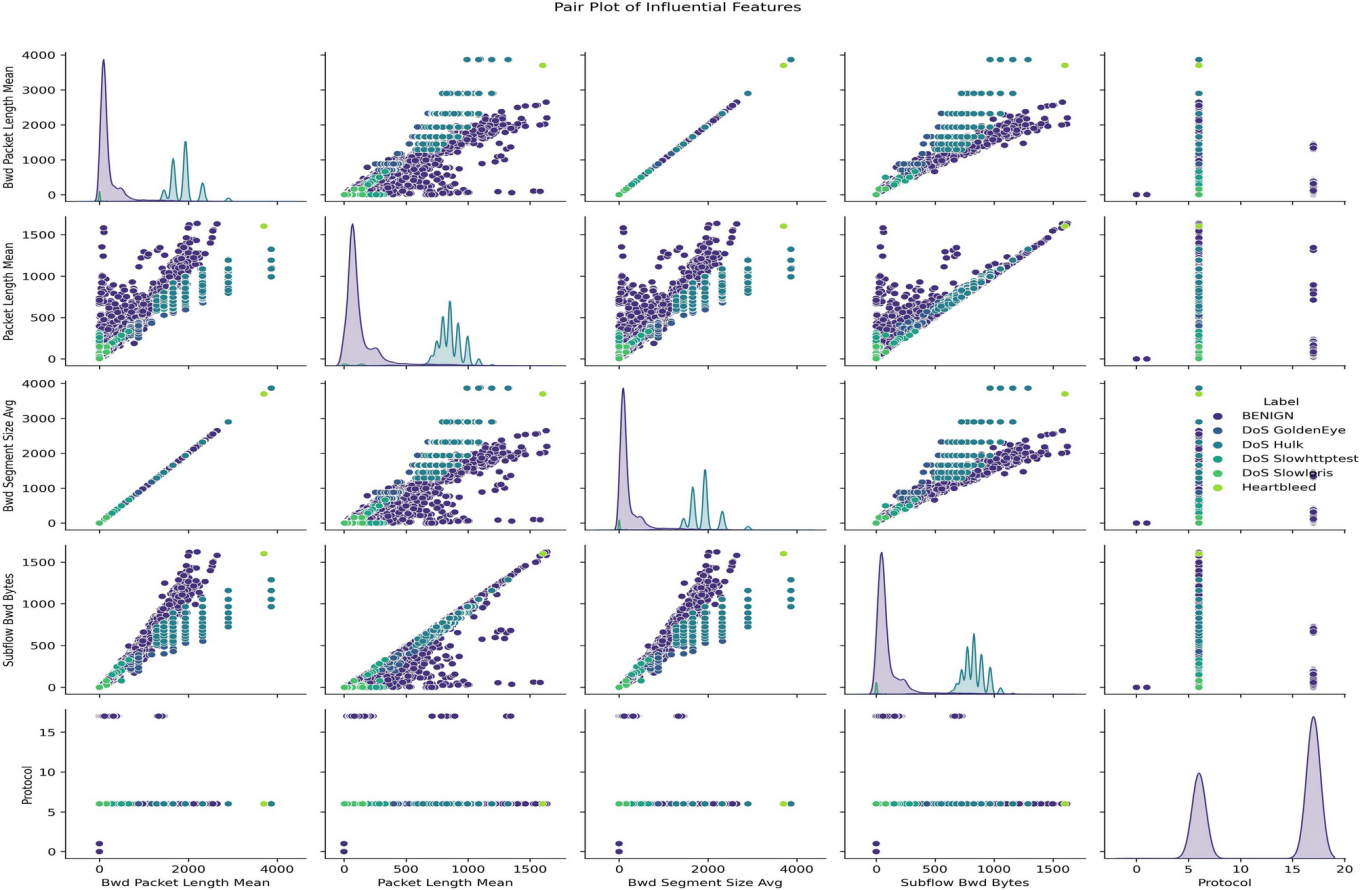
Pair plot of most influential features.

Figure 3 pair plot illustrates how feature relationships can help distinguish between attack types. The scatter plots show some clustering for specific attack classes, but the unequal distribution of points across classes reflects the underlying imbalance. The imbalance is further evidenced by the color-coded data points where particular attacks (e.g., DoS Hulk) dominate, and others are sparsely represented. This further supports the need for techniques like SMOTE to balance the dataset and ensure that the model does not favor majority classes.

Visualizing the dataset in these ways makes it more apparent that handling imbalance is essential for improving model performance, ensuring that both majority and minority classes are adequately represented in training.

## Methods

**Fig. 4:**
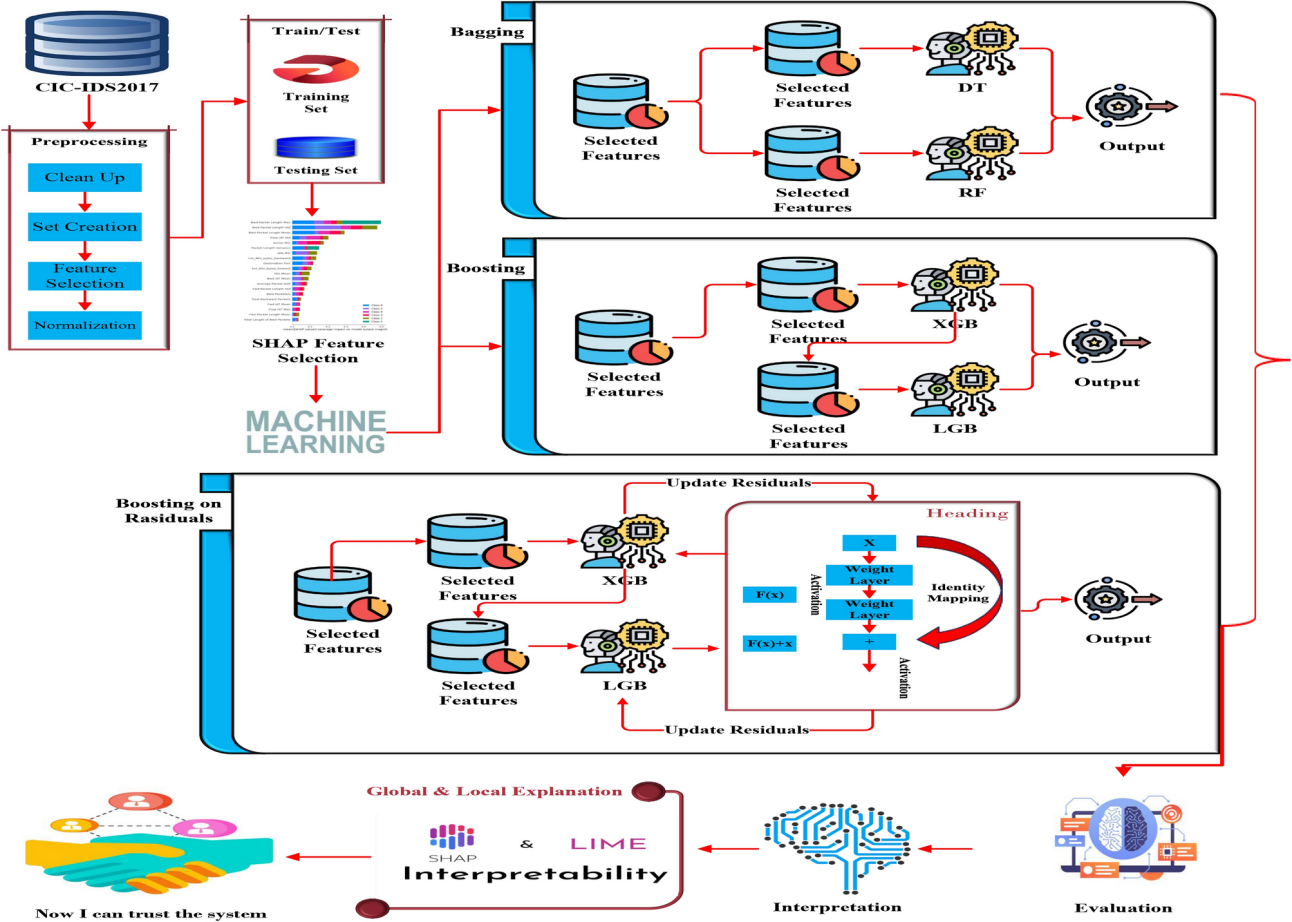
The proposed Hybrid bagging boosting residuals ensemble (HBB-RE) model.

This section presents the methodology using the procedures and techniques employed to reach the objectives as shown in Fig. 4.

### Data preprocessing

Preprocessing included handling missing values and class imbalance by applying SMOTE. The aim is to generate a clean, balanced dataset so that the built ML models have a level playing field and are likely effective, as shown in Table 2. Therefore, preprocessing is crucial to making the IDS more reliable and accurate. The CIC-IDS2017 dataset would make a strong candidate for developing advanced and explainable ML models for intrusion detection. More sophisticated methods are applied to missing values, such as imputing with the mean, median, or mode, and predictive models to impute the missing values.

**Table 2:** Dataset Preprocessing.

Preprocessing Step	
Data Cleaning	CIC-IDS2017
Missing Values	Replace NaN with mean/median
Duplicates	Remove duplicates
Outliers	Remove if $|z_i| > k$, $z_i = \frac{x_i - \mu }{\sigma }$
Feature Selection	
SHAP (DT)	Top 1000 features by SHAP values
Normalization	
Min-Max Scaling	$x_{ij}^{\prime } = \frac{x_{ij} - \min (x_i)}{\max (x_i) - \min (x_i)}$
Z-Score	$x_{ij}^{\prime } = \frac{x_{ij} - \mu _i}{\sigma _i}$
Categorical Variables	
One-Hot Encoding	Binary vector conversion
Label Encoding	Map labels to integers
Imbalanced Classes	
SMOTE	Generate synthetic samples
Without SMOTE	Alternative balancing
Oversampling	Duplicate minority samples
Undersampling	Reduce majority samples
Data Splitting	
Stratified Sampling	Preserve class distribution

### Model formulation

The detailed model formulation of the proposed model is as follows:

#### Bagging and boosting

Bagging (Bootstrap Aggregating) as shown in Algorithm 1 is a powerful ensemble method that improves the stability and accuracy of machine learning algorithms by reducing variance and helping to avoid overfitting. In the described approach, Bagging classifiers are initialized using DT and RF as the base estimators. Each base classifier is trained on a randomly selected subset of the training data, created by sampling with replacement (bootstrap sampling). Once the individual models are trained, the predictions from these models are averaged to produce a combined bagging prediction. This averaging helps smooth anomalies in individual model predictions, enhancing the model’s overall predictive performance and robustness.

Generate multiple bootstrap samples from the training data. Each sample is created by randomly selecting data points with replacements.

1 $$\begin{aligned} \{(x_i, y_i)\}_{i=1}^n \rightarrow \{(x_i^*, y_i^*)\}_{i=1}^n \end{aligned}$$

- $x_i$: Input data point at index *i*.
- $y_i$: Corresponding label for the input $x_i$.
- *n*: Total number of data points.
- $x_i^*$: Transformed input data point at index *i*.
- $y_i^*$: Transformed label for the input $x_i$.

##### Training base classifiers

Train a base classifier (DT, RF) on each bootstrap sample.

2 $$\begin{aligned} f_j(x) = \text {BaseClassifier}_j(x) \quad \text {for} \quad j = 1, 2, \ldots , B \end{aligned}$$

- $f_j(x)$: Prediction from the *j*-th base classifier for input *x*.
- $\text {BaseClassifier}_j$: The *j*-th base classifier model.
- *j*: Index of the base classifier, ranging from 1 to *B*.
- *B*: Total number of base classifiers.
- *x*: Input data for the classifiers.

Averaging Predictions: Combine the predictions from all base classifiers by averaging (for regression) or majority voting (for classification).

3 $$\begin{aligned} {\hat{y}} = \frac{1}{B} \sum _{j=1}^B f_j(x) \end{aligned}$$

- ${\hat{y}}$: Final aggregated prediction.
- *B*: Total number of base classifiers.
- $f_j(x)$: Prediction from the *j*-th base classifier for input *x*.
- *x*: Input data for the classifiers.
- $\sum _{j=1}^B$: Summation over all base classifiers from 1 to *B*.

Boosting is another ensemble technique that aims to create a robust classifier by sequentially combining several weak classifiers’ performances. Unlike Bagging, which focuses on reducing variance, Boosting focuses on reducing bias by iteratively correcting the errors made by previous models. First, an SGD-Classifier is trained using the residuals (errors) computed from the predicted probabilities of the first model. In other words, this model receives the actual values as target labels while the previously calculated predicted probabilities are subtracted and given as features. The resulting residuals are the component errors or deficiencies of the first model - that is, the areas where the predictions are more likely to be incorrect. Then, the models (XGBoost and LightGBM) are boosted sequentially on such residuals. Each model is trained to correct the errors of the previous model. After each training, predictions are recalibrated. This training gradually improves the model’s quality.

#### Boosting on residuals

The process starts with initial training on a linear, SGD-optimised classifier, predicting probabilities and calculating residuals, the errors or differences between the actual outcome and predicted probabilities.

After the initial training, Boosting is applied iteratively to the obtained residuals, as shown in the Algorithm 2. First, the XGBoost framework is trained on the residuals, learning how to correct the errors made by the SGD-Classifier; XGBoost is a fast and powerful gradient-boosting framework that has proven to be very versatile to tackle a wide variety of predictive problems. After training the XGBoost model on the residuals, the predictions are calibrated to reflect the newly acquired predictions. The procedure is then repeated on the residuals for another GB framework, LightGBM, another efficient, fast, and robust model that boasts superior performance over large datasets and behaves like an out-of-the-box regularizer on the problem at hand. With each iteration, the model predictions are refined to maximize accuracy by examining the areas where the previous models have underperformed, thereby progressively perfecting the model.

The detailed equation for boosting on residuals is as follows: Initial Model: Train the initial model $f_0$ using an SGDClassifier:

4 $$\begin{aligned} f_0(x) = \text {SGDClassifier}(x) \end{aligned}$$

- $f_0(x)$: Initial prediction model.
- $\text {SGDClassifier}(x)$: Stochastic Gradient Descent classifier applied to input *x*.
- *x*: Input data.

Residual Calculation: Calculate the residuals after each iteration. For the initial iteration, the residuals $r_i^{(1)}$ are calculated as:

5 $$\begin{aligned} r_i^{(1)} = y_i - f_0(x_i) \end{aligned}$$

- $r_i^{(1)}$: Residual for the *i*-th data point after the initial model.
- $y_i$: True label for the *i*-th data point.
- $f_0(x_i)$: Initial prediction for the *i*-th data point.
- $x_i$: Input data point at index *i*.

Boosting Iterations: For each subsequent iteration $m$:

6 $$\begin{aligned} r_i^{(m)} = y_i - {\hat{y}}_i^{(m-1)} \end{aligned}$$

- ${\hat{y}}_i^{(m-1)}$ is the prediction from the model
- $$m-1$$ iterations.

Train the new model $h_m$ (XGBoost or LightGBM) on the residuals:

7 $$\begin{aligned} h_m = \text {XGBoost/LightGBM}(r_i^{(m)}) \end{aligned}$$

- $h_m$: *m*-th weak learner or booster model.
- $\text {XGBoost/LightGBM}$: Model using either XGBoost or LightGBM applied to residuals.
- $r_i^{(m)}$: Residual for the *i*-th data point in the *m*-th iteration.

Update the Model: Update the model by adding the new model’s prediction:

8 $$\begin{aligned} {\hat{y}}_i^{(m)} = {\hat{y}}_i^{(m-1)} + \lambda h_m(x_i) \end{aligned}$$

- ${\hat{y}}_i^{(m)}$: Predicted value for the *i*-th data point after *m* iterations.
- ${\hat{y}}_i^{(m-1)}$: Predicted value for the *i*-th data point after $m-1$ iterations.
- $\lambda$: Learning rate for model updates.
- $h_m(x_i)$: *m*-th weak learner prediction for the *i*-th data point.

Final Model: The final model after $M$ iterations is:

9 $$\begin{aligned} F_M(x) = f_0(x) + \sum _{m=1}^{M} \lambda h_m(x) \end{aligned}$$

- $F_M(x)$: Final prediction after *M* iterations.
- $f_0(x)$: Initial prediction model.
- $\sum _{m=1}^{M}$: Summation over all *M* boosting iterations.
- $\lambda$: Learning rate for model updates.
- $h_m(x)$: *m*-th weak learner’s prediction for input *x*.
- *M*: Total number of boosting iterations.

At the end of this iterative boosting process, the final predictions are obtained by converting the combined probabilities back to binary form-making sure that the result of all the boosting stages improves the original classification problem. The combination of Bagging and Boosting on residuals leverages the strengths of both techniques, resulting in a robust and highly accurate predictive model.

### Proposed HBB-RE models

The study proposes an integrated approach, the hybrid method HBB-RE, to effectively improve the IDPS by first integrating two ensemble learning techniques, bagging and boosting, to capitalize on their strengths while mitigating their weaknesses, enhancing performance and interpretability. Bagging works by training an ensemble of models in parallel on different subsets of the original data obtained by random sampling with replacement. In other words, this will reduce the variance and prevent overfitting since it averages the predictions generated by the various models to yield a robust initial model representing diverse patterns in the data.

**Algorithm 1:**
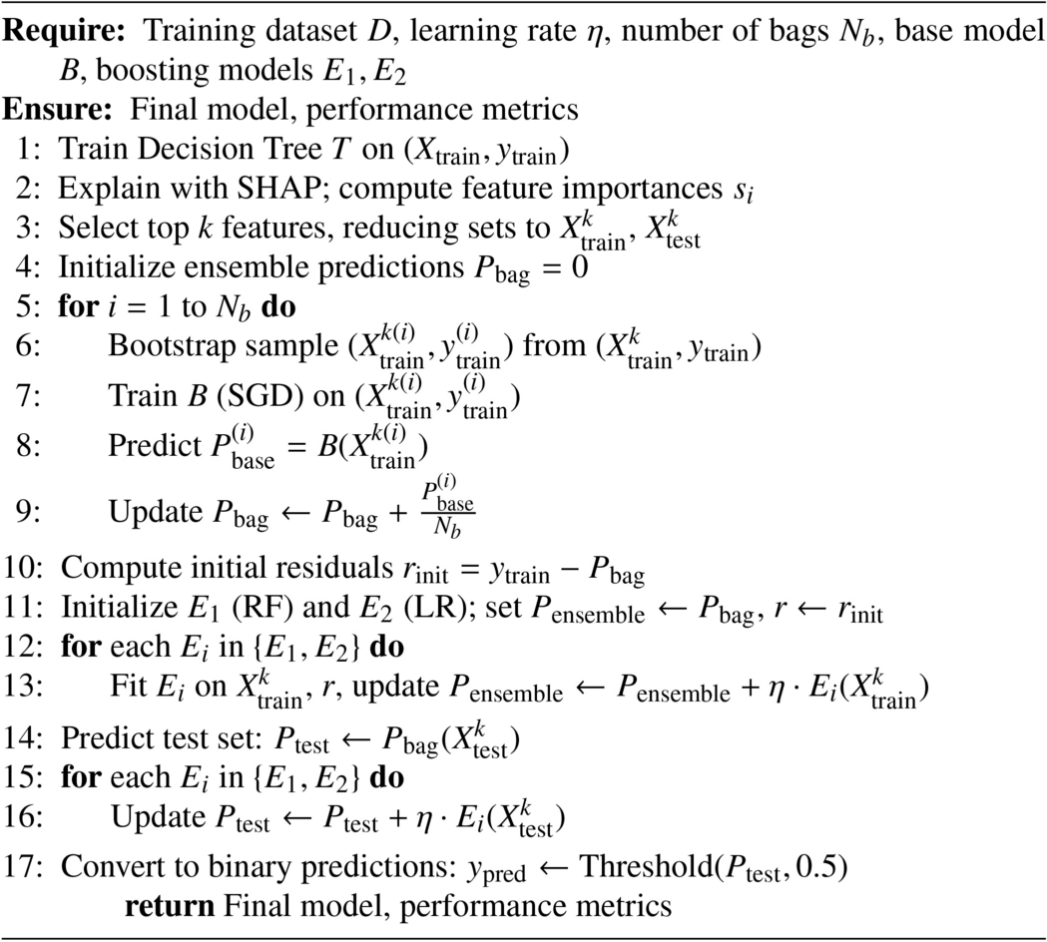
Hybrid Bagging and Boosting

It applies to boost the residual errors finally after the bagging method. The residuals are the errors or differences between the actual values and the predictions made by the initial model of bagging. Boosting will focus on these residuals by training new models sequentially, each trying to correct its predecessor’s mistakes. It reduces bias in the model by putting more weight on boosting misclassified instances, giving the model overall better accuracy.

The combination of bagging and boosting enables HBB-RE to handle both problems of variance and bias; therefore, it generalizes and performs better on unseen data. The method also points to the SHAP values in feature selection and interpretability. The SHAP values highlight the most informative features, further making the model more transparent and unveiling different features in the contribution toward the prediction.

The HBB-RE approach gives an organized method for intrusion detection by adding ensemble techniques to feature interpretability tools. This hybrid strategy will improve intrusion detection performance and provide important insight into the model’s decision-making process, which is imperative in practical IDPS applications.

SHAP provides better performance and interpretability than other popular feature selection methods. While RFE provides good accuracy by iteratively removing less essential features, it lacks the capability of SHAP to explain individual feature contributions from a global and local view. LASSO regularization effectively penalizes dimensionality by setting some coefficients to zero, which improves model performance; however, it cannot quantify the exact impact of each feature on predictions, as SHAP does.

**Algorithm 2:**
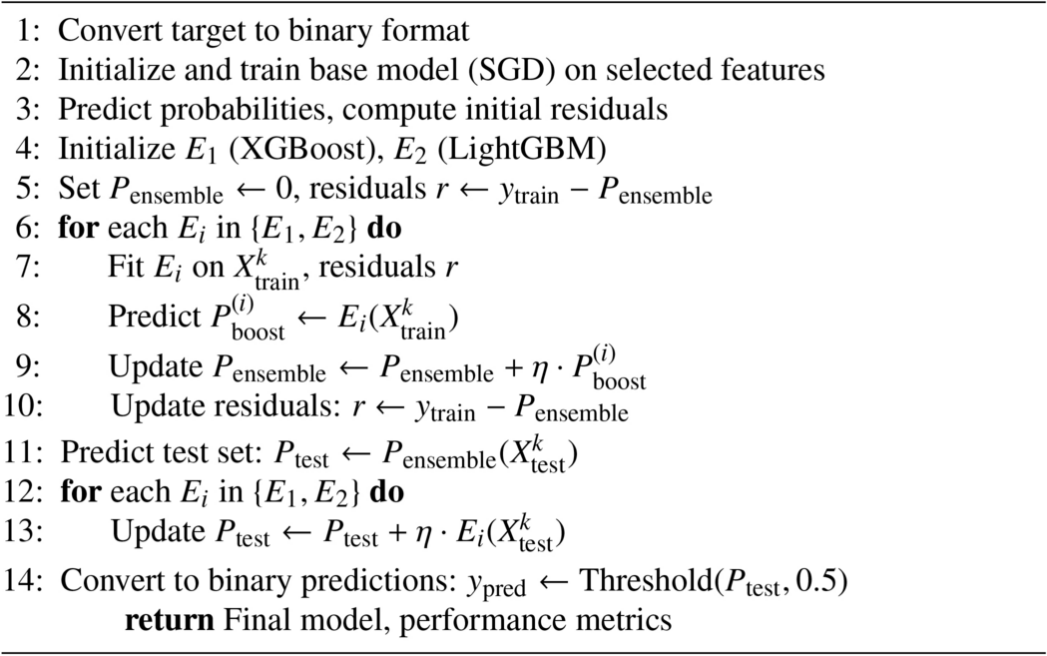
Boosting on Residuals

Although efficient in reducing multicollinearity, PCA transforms features into principal components, making it less interpretable and challenging to map back to original features. Unlike the rest, SHAP identifies which features are essential and explains whether they contribute positively or negatively to individual predictions, making it a complete tool for feature selection.

By including SHAP in this approach, the aim is to complete a model that performs well and is interpretable by intrusion detection. Using SHAP allows for detailed interpretability of feature effects, enabling one to understand how the model makes its decisions, which is crucial for effective and reliable security systems.

### Evaluation matrices

After carefully categorizing data points, conventional measures like accuracy^35^,

10 $$\begin{aligned} \text {Accuracy} = \frac{\text {Number of Correct Predictions}}{\text {Total Number of Predictions}} \end{aligned}$$

- Correct Predictions: Matches with actual labels.
- Total Predictions: All predictions made.

F1-score,

11 $$\begin{aligned} \text {F1} = 2 \times \frac{\text {Prec} \times \text {Rec}}{\text {Prec} + \text {Rec}} \end{aligned}$$

- Prec: TPs / Pred Ps.
- Rec: TPs / Act Ps.

Classification reports,

12 $$\begin{aligned} \text {Precision} = \frac{\text {TPs}}{\text {TPs} + \text {FPs}} \end{aligned}$$

- TPs: Correctly predicted Ps.
- FPs: Incorrectly predicted Ps.

And ROC curves are used to evaluate the overall performance.

13 $$\begin{aligned} \text {Recall} = \frac{\text {TPs}}{\text {TPs} + \text {FNs}} \end{aligned}$$

- TPs: Correctly predicted Ps.
- FNs: Missed actual Ps.

14 $$\begin{aligned} \text {AUC} = \int _{0}^{1} \text {ROC}(t) \, dt \end{aligned}$$

- AUC: Area under ROC curve.
- ROC(*t*): ROC value at threshold *t*.

This step provides valuable insights into the performance of each model across several classes. The evaluation report includes each model’s efficiency and distinguishes between different classes. A cross-validation method was applied to evaluate the model performance. Using k-fold cross-validation^36^ with 5-fold, the models’ accuracy was assessed by consistency and generalizability across different training data splits.

15 $$\begin{aligned} \text {CV Acu} = \frac{1}{k} \sum _{i=1}^{k} \text {Acu}_i \end{aligned}$$

- *k*: Number of cross-validation folds (5).
- $\text {Accuracy}_i$: Accuracy for the *i*-th fold.
- $\frac{1}{k} \sum _{i=1}^{k}$: Average accuracy across all *k* folds.
- $k-1$: Number of parts used for training in each fold.

### Implementation details

For building a robust system for ML intrusion detection, a system with these specifications is used as shown in Table 3.

**Table 3:** System Specifications.

System	Description
CPU	AMD Ryzen 9 for efficient multi-threaded processing.
GPU	NVIDIA RTX 3080 with CUDA acceleration.
RAM	32GB DDR4 for handling large datasets.
Storage	512GB NVMe SSD + 500GB HDD for data storage.
Cooling System	High-quality cooling system for optimal performance.
Power Supply	750W Gold certified for efficiency and stability.
Operating System	Windows 10 Professional Edition.
Programming Language	Python for ML/DL.
ML Libraries	TensorFlow, PyTorch, scikit-learn.
Data Processing	Pandas, NumPy for manipulation.

This setup balances computational power, speed, and storage, allowing for efficient training and deployment of ML/DL models.

## Results and discussion

The result section demonstrates the proposed model’s efficacy in predicting reasonably accurate outcomes across various test scenarios. Key performance metrics, including ROC-AUC, precision, and SHAP-based feature importance, reveal much about model behavior and predictive power.

### Confusion matrix with SMOTE

This section presents the performance of a binary and multiclass classification model applied to the CIC-IDS2017 dataset, with the (SMOTE) to address the class imbalance.

**Fig. 5:**
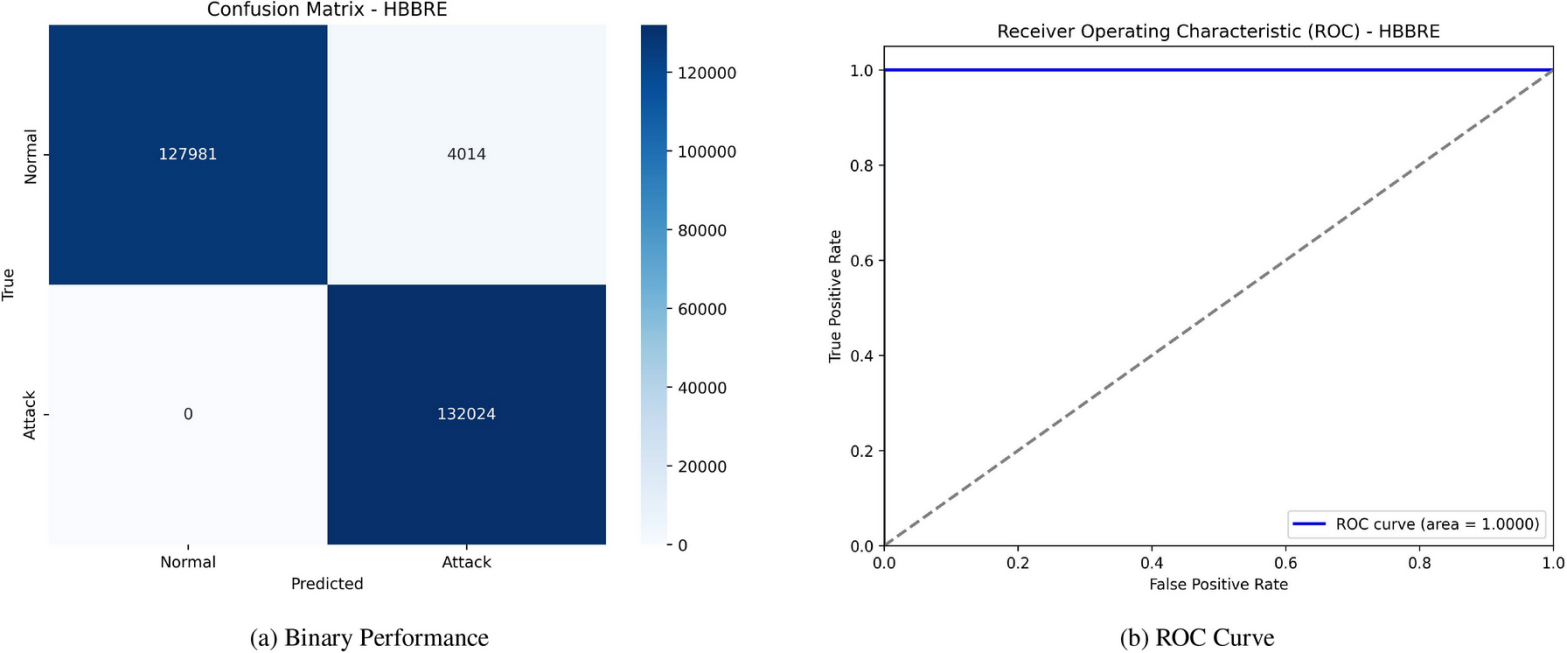
Confusion matrices and ROC curves with SMOTE.

**Fig. 6:**
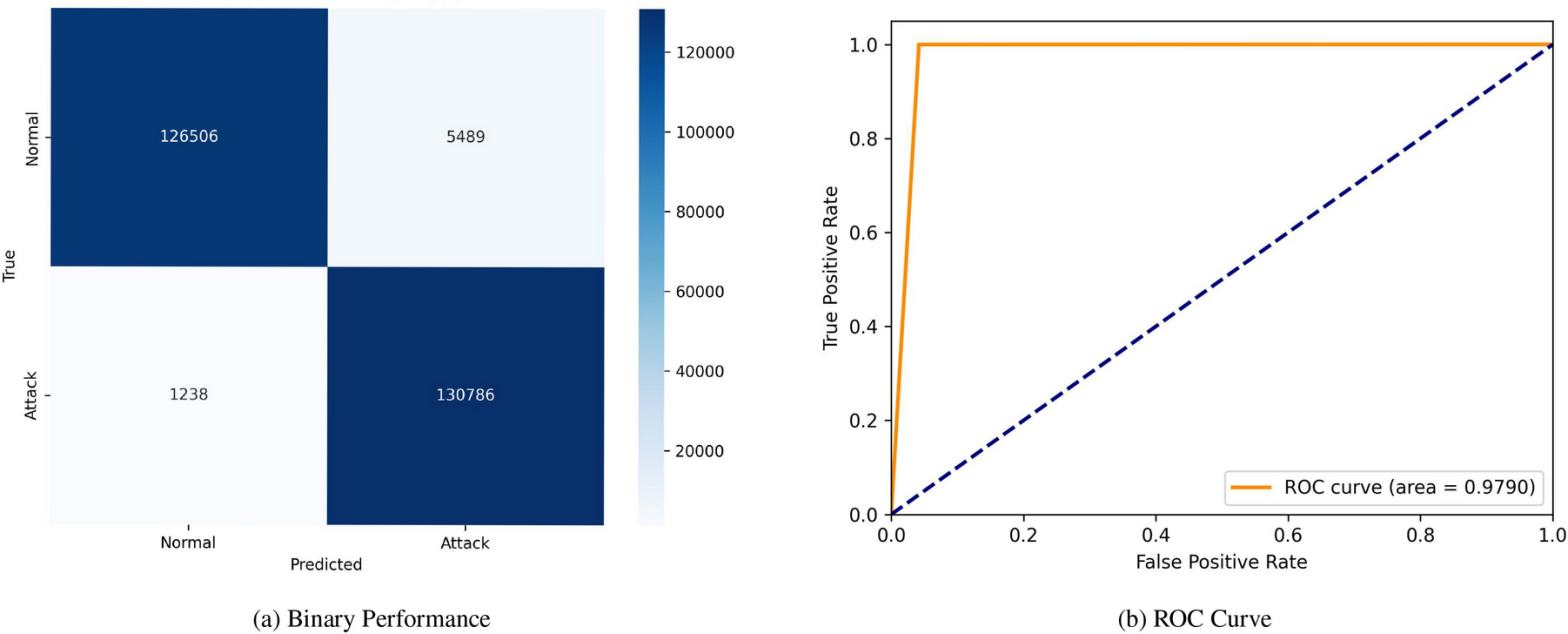
Confusion matrices and ROC curves with SMOTE.

Figure 5 displays the proposed model’s confusion matrix and (ROC) curve. The confusion matrix reveals high accuracy, with 127,981 TN and 132,024 TP, alongside 4,014 FP and no FN. The (ROC) curve corresponding to this performance showcases an area under the curve (AUC) of 1.0000, indicating perfect class discrimination. Figure 6 similarly presents the proposed model’s confusion matrix and (ROC) curve under different conditions or possibly another model iteration. In this case, the confusion matrix records 126,506 TN, 130,786 TP, 5,489 FP, and 1,238 FN. This setup’s (ROC) curve shows a slightly lower (AUC) of 0.9790, signifying a minor decline in classification performance.

**Fig. 7:**
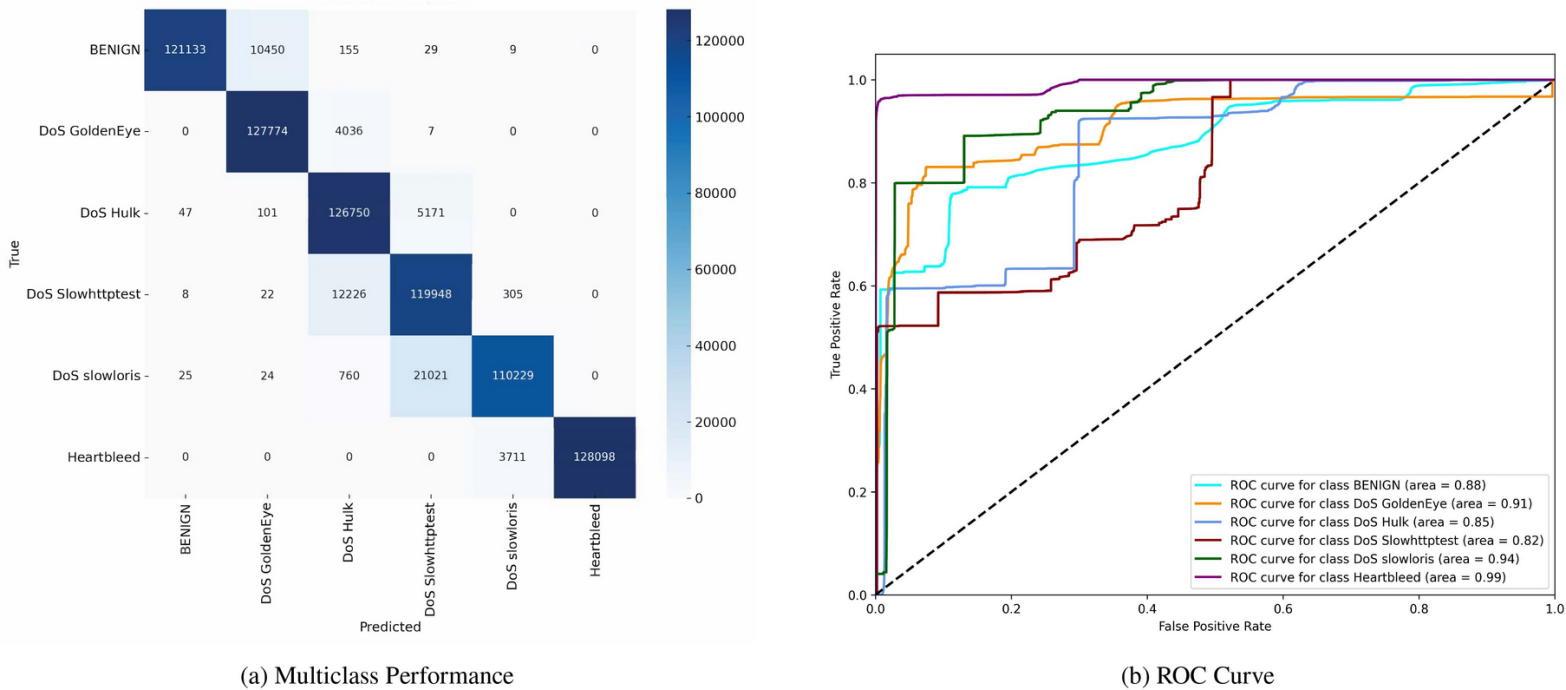
Confusion matrices and ROC curves with SMOTE.

Figure 7 shows the confusion matrix and the (ROC) curves for various classes in the dataset. The confusion matrix indicates the model’s ability to distinguish between multiple classes, including ’BENIGN,’ ’DoS GoldenEye,’ ’DoS Hulk,’ ’DoS Slowhttptest,’ ’DoS slowloris,’ and ’Heartbleed.’ The matrix highlights TP and misclassification, such as 121,133 TN for ’BENIGN’ and 127,774 TP for ’DoS GoldenEye,’ and a notable number of misclassified instances across other classes. The (ROC) curves display the model’s performance for each class, with (AUC) values ranging from 0.82 to 0.99, suggesting varying degrees of classification accuracy among the different types of network traffic.

**Fig. 8:**
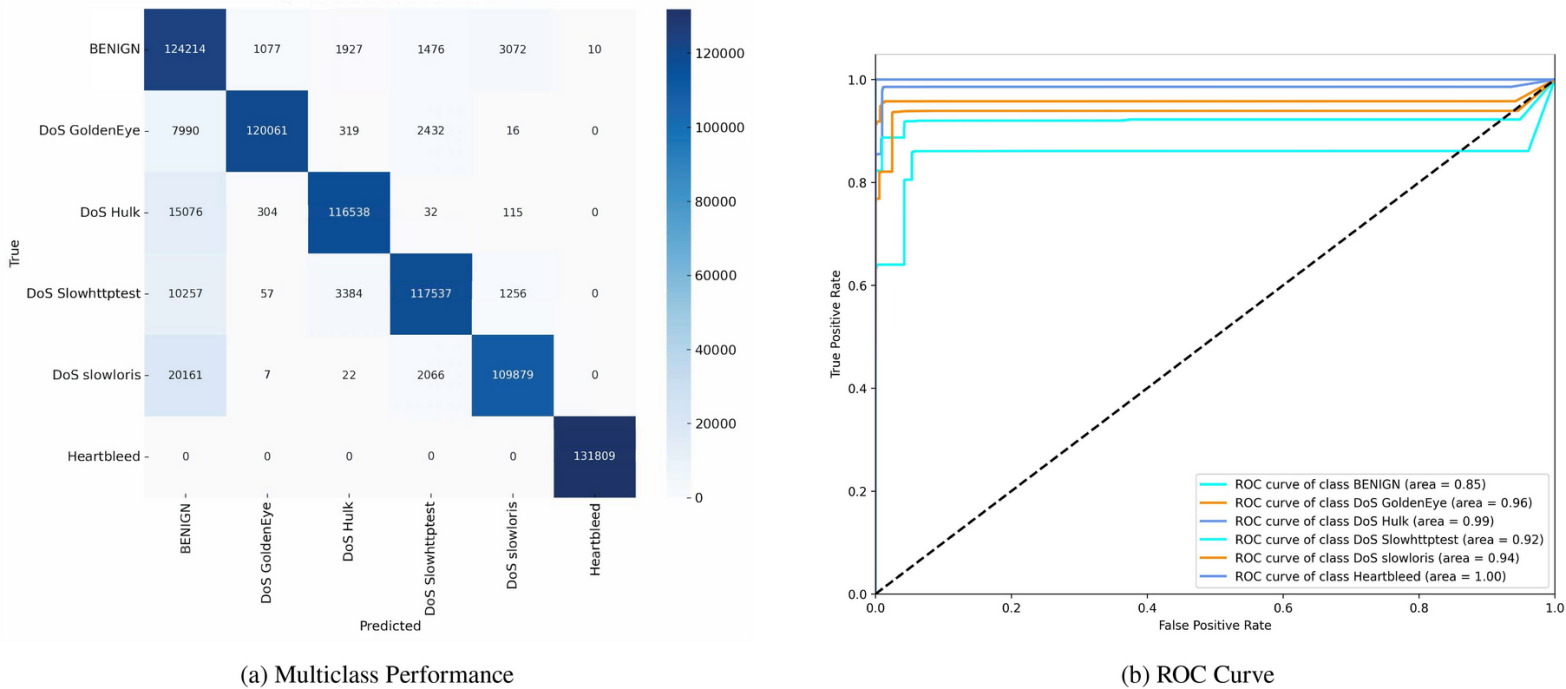
Confusion matrices and ROC curves with SMOTE.

Figure 8 similarly depicts the confusion matrix and (ROC) curves, reflecting another evaluation of the same multiclass classification model. The confusion matrix demonstrates the distribution of correctly and incorrectly classified instances, with 124,214 TN for ’BENIGN’ and 121,061 TP for ’DoS GoldenEye.’ Mis-classifications are also noted, particularly within ’DoS Hulk’ and other attack types. The (ROC) curves for this evaluation indicate (AUC) values between 0.85 and 1.00, underscoring a solid classification performance overall, although some classes exhibit lower discriminative power.

### Confusion matrix without SMOTE

This section presents the performance of a binary and multiclass classification model applied to the CIC-IDS2017 dataset without the (SMOTE) to address the class imbalance.

**Fig. 9:**
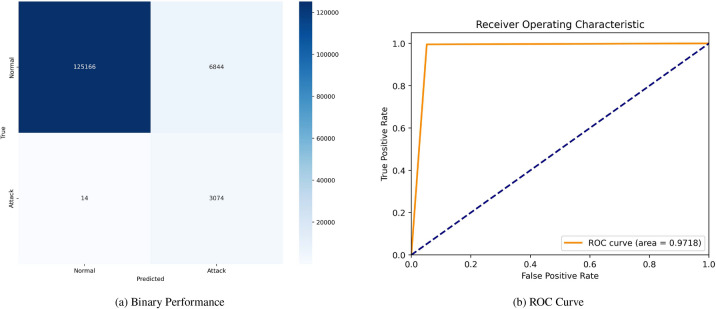
Confusion matrices and ROC curves without SMOTE.

**Fig. 10:**
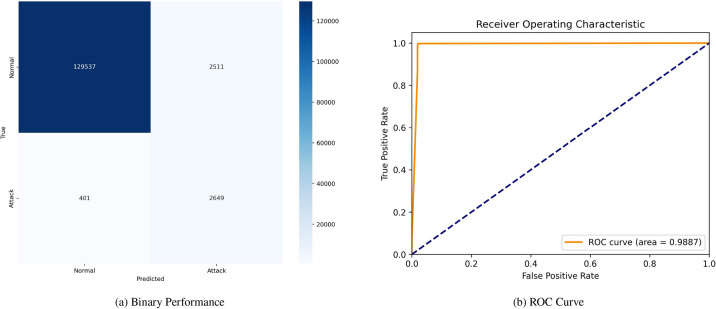
Confusion matrices and ROC curves without SMOTE.

Figure 9 showcases the confusion matrix and (ROC) curve for the model. The confusion matrix shows 125,166 TN and 3,074 TP, with 6,844 FP and 14 FN. The (ROC) curve exhibits an area under the curve (AUC) of 0.9718, indicating overall solid performance despite the absence of SMOTE. Figure 10 also presents the confusion matrix and (ROC) curve for the model. The confusion matrix reports 129,537 TN and 2,649 TP, alongside 2,511 FP and 401 FN. The corresponding (ROC) curve has a slightly higher (AUC) of 0.9887, demonstrating improved discriminative ability.

**Fig. 11:**
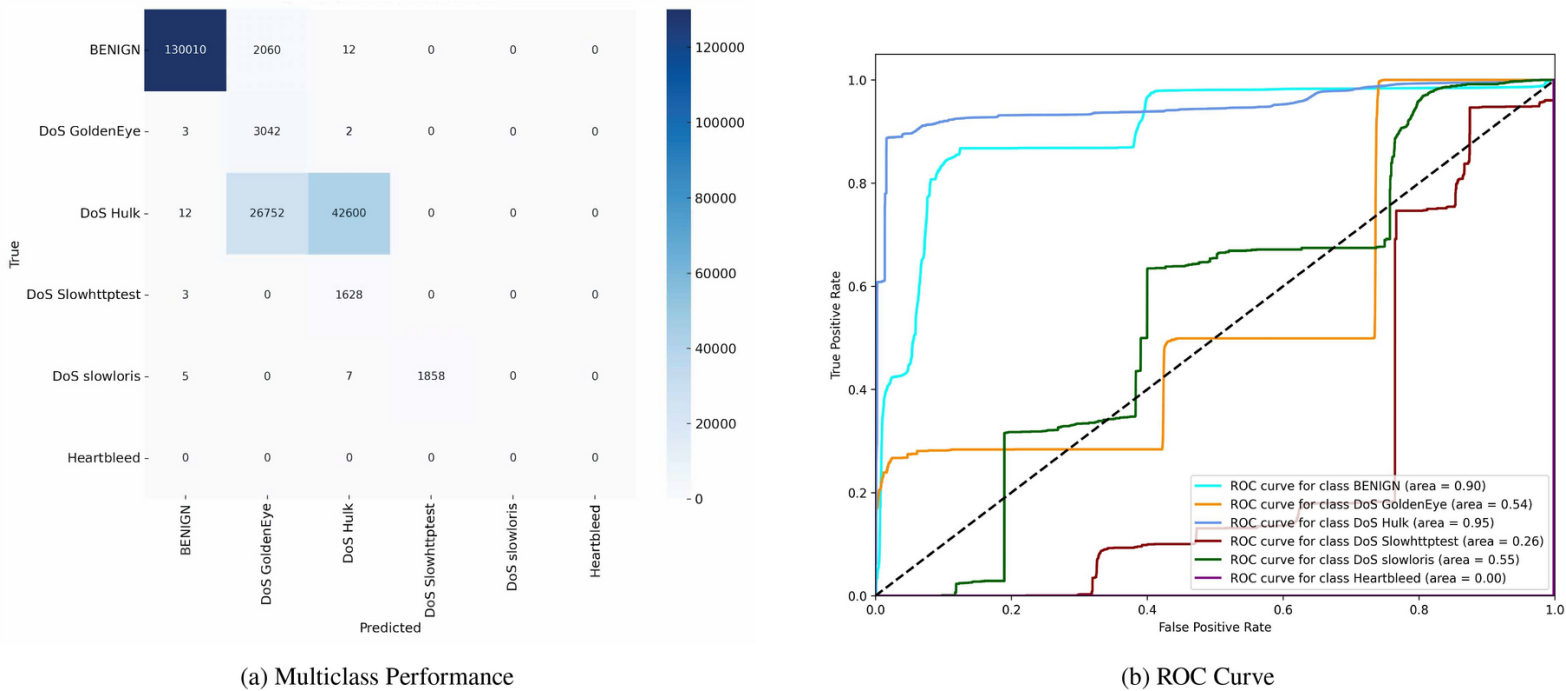
Confusion matrices and ROC curves without SMOTE.

Figure 11 reveals that while the model robustly classifies ’BENIGN’ traffic, evidenced by 130,010 TP, there are notable mis-classifications, particularly with ’DoS Hulk’ attacks, which show many FN (42,060). The corresponding (ROC) curves reflect the model’s varying ability to distinguish between different attack types, with the (AUC) for ’BENIGN’ traffic at 0.950, indicating high discriminative power, whereas ’DoS Slowloris’ and ’DoS Slowhttptest’ have lower (AUC) values of 0.31 and 0.26, respectively, indicating poorer performance in these classes.

**Fig. 12:**
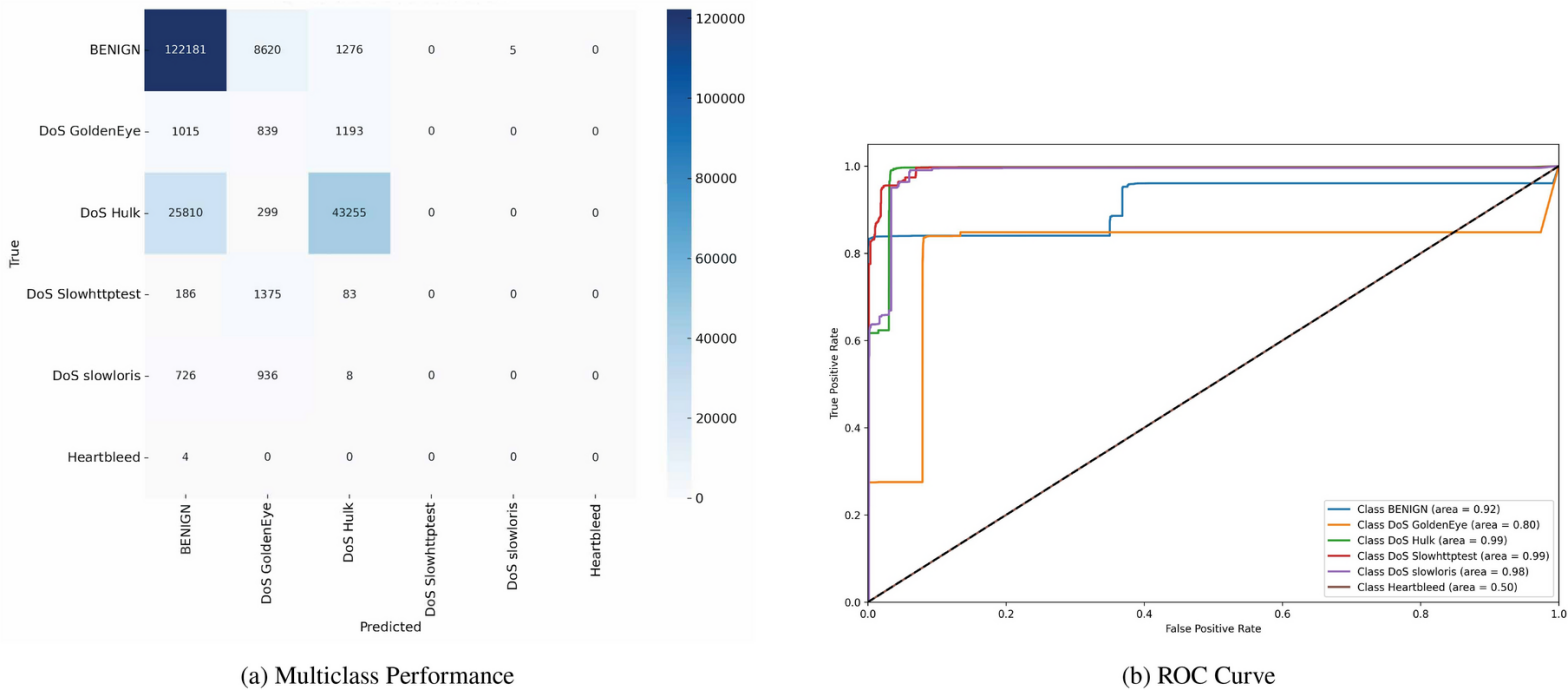
Confusion matrices and ROC curves without SMOTE.

Figure 12 shows improved detection for underrepresented attack types, such as ’DoS Hulk’, with an increased TP count of 29,180 and reduced FN (42,353). The (ROC) curves corroborate this improvement, showing enhanced (AUC) values across most classes, notably ’BENIGN’ (0.930) and ’DoS Hulk’ (0.930). However, some attack types still exhibit challenges in classification, as indicated by ’DoS Slowhttptest’ ((AUC) of 0.91) and ’Heartbleed’ ((AUC) of 0.860).

Despite the robust performance, the confusion matrices reveal misclassifications, including normal instances predicted as attacks. In contrast, FPs and FNs indicate areas for improvement in the model’s predictions for binary and multiclass classifications.

A high peak in accuracy and the F1 score for the model is promising; however, for real-world IDPS, this requires more elaborate interpretation. High accuracy means the model is generalized well to most normal and attacks class instances correctly. However, in the case of real-world IDPS, accuracy alone cannot be deemed appropriate because of the class imbalance problem; a high number of instances from normal traffic could skew the results, and therefore, precision and Recall are more essential metrics on which to focus.

In practice, the performance measure of interest in IDPS is the F1 score. A higher F1 will indicate that most of the attacks have been effectively detected by the model with high Recall. At the same time, it generates few false alarms (high precision), which is very important for real-world applications since a high rate of false positives would result in unnecessary interventions, increasing the system overhead and the operational cost of such systems. On the other hand, false negatives may tend to allow hidden attacks to compromise network security.

The high F1 score, therefore, reflects the model’s potential to be reliable in intrusion detection and ensure that operation efficiency is maintained; thus, it is more applicable on a wide scale of real-world deployment. In other words, while the metrics look promising, their interpretation underlines IDPS as a model that could balance the detection and false alarm rate. It means more realistic, efficient, effective, and practical intrusion detection.

Now, extending the HBB-RE model for multiclass classification involves several areas of significant modification necessary to remain robust while managing the added complexities of multiclass settings. While initially designed for binary classification, the HBB-RE was extended using a hierarchical strategy. Two successive classifications were done to further refine class discrimination in iterative steps. It first carries out binary classification between normal traffic and suspicious activities, followed by several one-vs-rest classifications that identify specific attack types.

Several ensemble model components had to be carefully tuned to implement this multiclass strategy, paying particular attention to residual boosting so that residual errors from one class would not combine cascadingly or excessively bias the identification of other classes. Also, applying SHAP for feature selection to highlight such relevant features at each stage in classification helps in the model’s decision-making process and interpretability across the hierarchy.

The challenges for this extension mainly revolved around handling the class imbalance, where attack types were fewer within some classes, making it very tough to train a classifier to handle them. Further, propagating errors may occur because early-stage misclassifications affect downstream multiclass predictions. These are mitigated by integrating resampling techniques and fine-tuning hyperparameters for each class.

This extension has shown the ability to generalize across different attack types using HBB-RE. Additionally, this work has provided insight into adapting the ensemble models for multiclass intrusion detection, with which further research can extend scalability and precision in real-world IDPS.

The results of the binary and multiclass classification models for the CIC-IDS2017 dataset, with/without the SMOTE application, show their high accuracy (AUC) and robust classification metric scores. These results show that the models are discriminatory, especially regarding different attack types. However, the Confusion Matrices show that although these models are very effective, there is still a lot of room for improvement in the future. Refine these models to improve their predictive ability to reduce errors such as FPs and FNs. These models have the potential to contribute to the early detection and prevention of intruders on the network, thereby providing a network environment that is safer and more reliable.

### Learning curve analysis

**Fig. 13:**
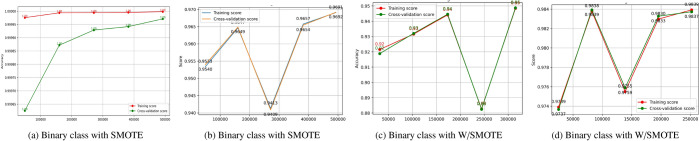
Learning curve for binary class with/without SMOTE.

Figure 13 illustrates learning curves comparing binary classification performance with/without SMOTE. Panels (a) and (b) show the results with SMOTE applied, while panels (c) and (d) represent models trained without SMOTE. Panel (a) features high training accuracy and an upward trend in cross-validation accuracy as the sample size increases, indicating better model generalization after SMOTE. While there are minor fluctuations in panel (b) for the cross-validation score, this may hint at the model’s sensitivity towards changes in data and, hence, be used to get an intuition about which directions the model could further be optimized. Panels (c) and (d) show models without applying SMOTE. For panel (c), the mid-range value for cross-validation accuracy indicates that specific tuning would be required to handle the original distribution of the data. On the other hand, panel (d) also reflects a consistent trend between training and cross-validation scores, confirming that class weighting alone may be adequate to support model generalization in the case of some problems. These observations highlight how SMOTE and class weighting can help the stability and performance generalization of models across different approaches in data handling.

**Fig. 14:**
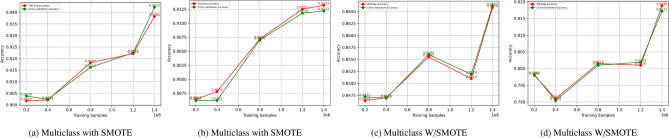
Learning curve for multiclass with/without SMOTE.

Figure 14 presents learning curves for multiclass classification for SMOTE with and without weighting in each panel. Panels (a) and (b) show the results with the application of SMOTE: in panel (a), the training and cross-validation accuracies steadily rise as one increases the number of training samples and converge to approximately 0.92, which indicates that good generalization is achieved with SMOTE for multiclass classification. Panel (b) suggests that both measures increase monotonically to reach near convergence at 0.91, indicating that SMOTE allows for stable learning across classes. Without SMOTE but with weighting, panels (c) and (d) present the following: in panel (c), accuracy stabilizes around 0.86, with minor differences between training and cross-validation, suggesting balanced generalization even without SMOTE. Panel (d), on the other hand, shows lower and spikier performance, with stabilization after a while at around 0.82; this may show some problems with capturing minority classes without SMOTE. Overall, from these results, it would appear that these multiclass problems improve the model’s performance and generalization while weighting is moderately successful; however, it is more sensitive to data imbalancedness.

### Explainable AI analysis

Explainable AI makes machine learning behavior and predictions understandable to humans-a different methodology and technique^37^. Traditional artificial intelligence models can be highly performant, but at the same time, they are considered black boxes: hard to understand and the reason behind making decisions^38^. This understanding is a significant problem in those domains where trust and accountability are crucial-such as cybersecurity^39,40^. Explainable AI would become helpful in giving an essential reason for making those models and indicating which features or patterns have led to which kinds of conclusions. Such transparency is crucial for verifying the model’s reliability, possibly diagnosing biases, and ensuring that it complies with the domain knowledge and requirements to which it will be applied^41^.

LIME visualization allows for an in-depth analysis of how the model processes individual data points^42^, which is crucial for model validation and debugging and in cases where model interpret-ability is necessary, such as in domains with regulatory requirements explained^43^ to stakeholders.

### Limitations of existing methods

The critical milestone toward integrating Explainable AI techniques, such as SHAP feature selection, into cybersecurity is that intrusion detection through traditional methods tends to be “black boxes.” Lack of insight into the decision-making process presents challenges in understandability, trust, and compliance in environments that require accountability.

Since these are shortcomings of existing systems, the proposed approach throws more light on which features are most responsible for detecting cyber threats by incorporating SHAP feature selection. This will enhance model interpretability and allow security analysts to understand which indicators of malicious activity are most relevant. Moreover, it develops a better relationship wherein analysts can be more trustful of system decisions, monitor more effectively, and respond faster.

Apart from that, model complexity would be positively affected with the application of SHAP, as feature selection for the most impactful improves the computational efficiency level without performance sacrifice. This is particularly important for intrusion detection systems where speed is essential in real-time. Explainability will also enable compliance with various sets of regulations on transparency within automated decision-making processes.

In other words, integrating Explainable AI techniques such as SHAP directly tackles the shortcomings of current cyber security approaches by improving intrusion detection systems’ transparency, feature selection, and intrusiveness.

### SHAP analysis

The SHAP analysis shows the impact of features on the binary and multiclass classification performance using SHAP values for Bagging and Boosting residual models with and without using SMOTE.

#### SHAP summary plot

**Fig. 15:**
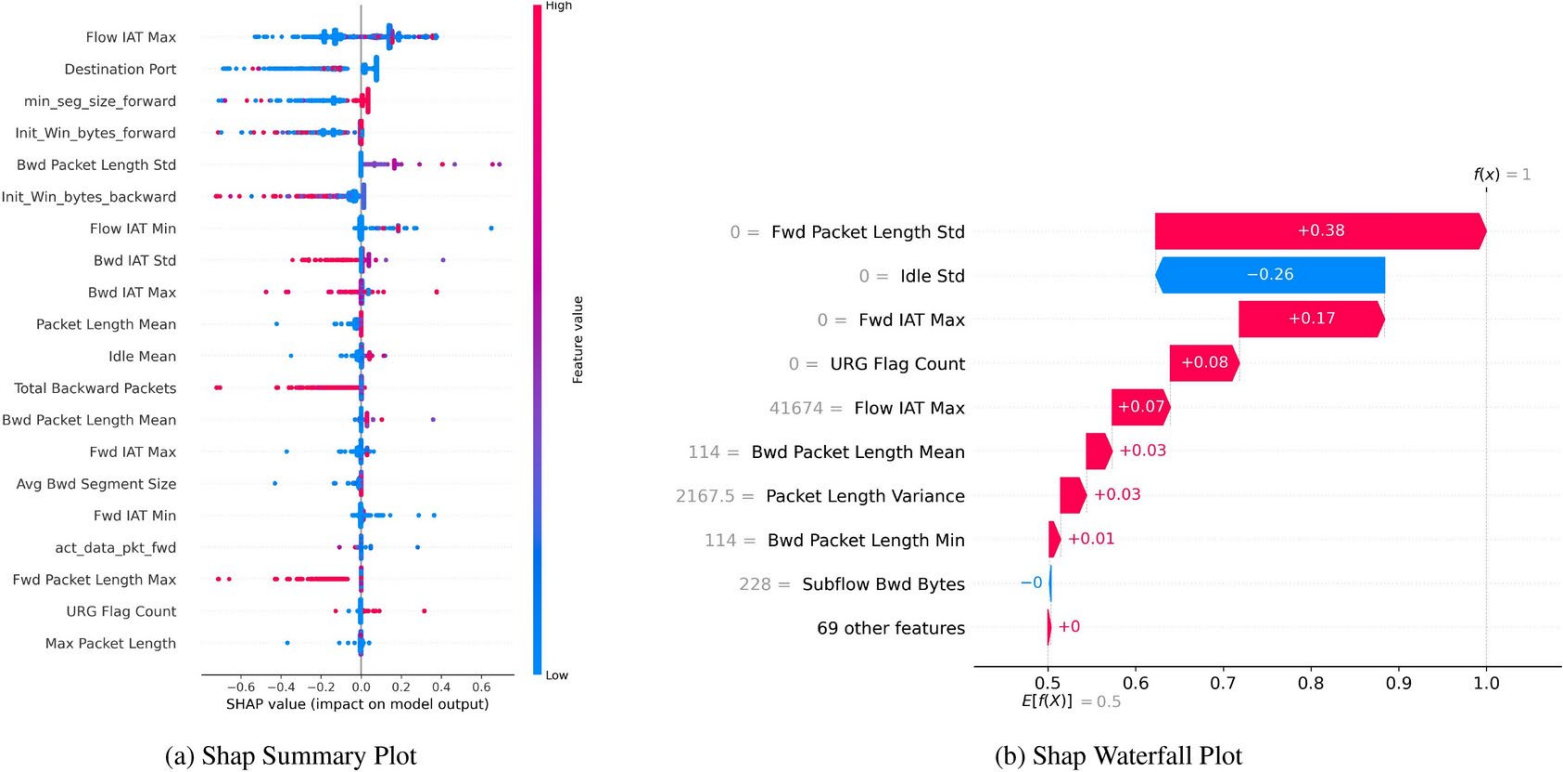
SHAP with SMOTE indicating robust bagging and boosting binary classification performance.

Figure 15 highlights the contribution of each feature via the SHAP Summary Plot Fig. 15a and SHAP Waterfall Plot Fig. 15b to reach the final result for a particular instance. The colored dots in the summary plot represent the SHAP value of each feature, which determines how the feature influences the model’s output, solidly depicted at the end. You can see that features are sorted in descending order according to their relevance. Another observation is that the color bar for each feature identifies if its value is high or low. For instance, the feature ’Flow IAT Max’ significantly affects the model prediction when its value is too high.

**Fig. 16:**
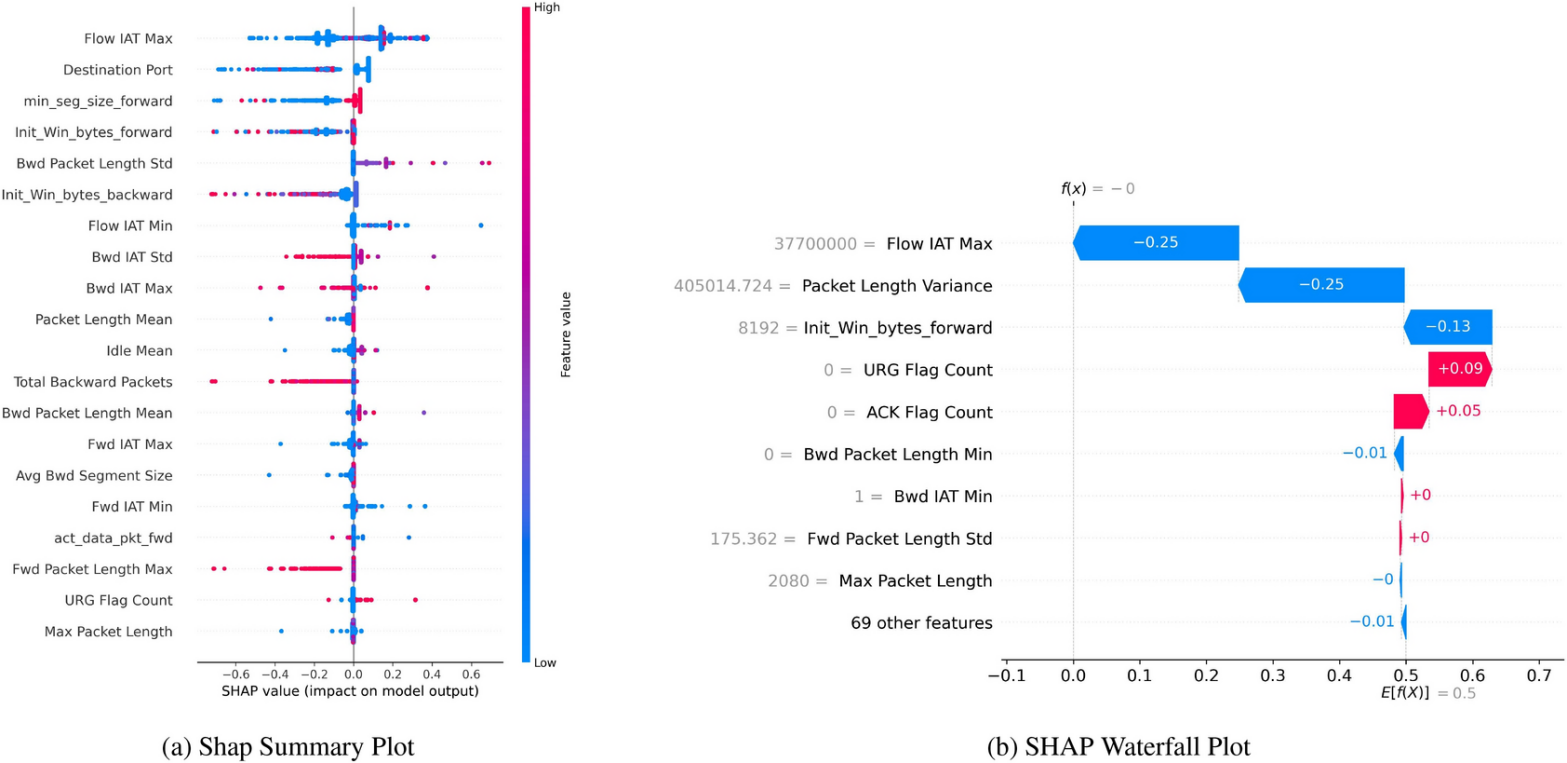
SHAP with SMOTE, indicating robust boosting of residuals’ binary classification performance.

Figure 16 gives an insight into the Shap feature values. The Shap Summary Plot Fig. 16a represents the features in descending order of importance with their Shap values and a bar indicating the contribution of the value of the SHAP feature to the model output. In the model, the “Flow IAT Max” has the most significant impact on the class classification of the attack, with Shap values ranging from approximately -0.6 to 0.6, which is a higher range as compared to all other features and depicts the impact of the feature on the predictions, with higher values contributing to higher predictions (red color). Other significant features that contribute both positively and negatively to classification are “Destination Port”, “min seg size forward”, and “Init Win bytes forward”, respectively.

**Fig. 17:**
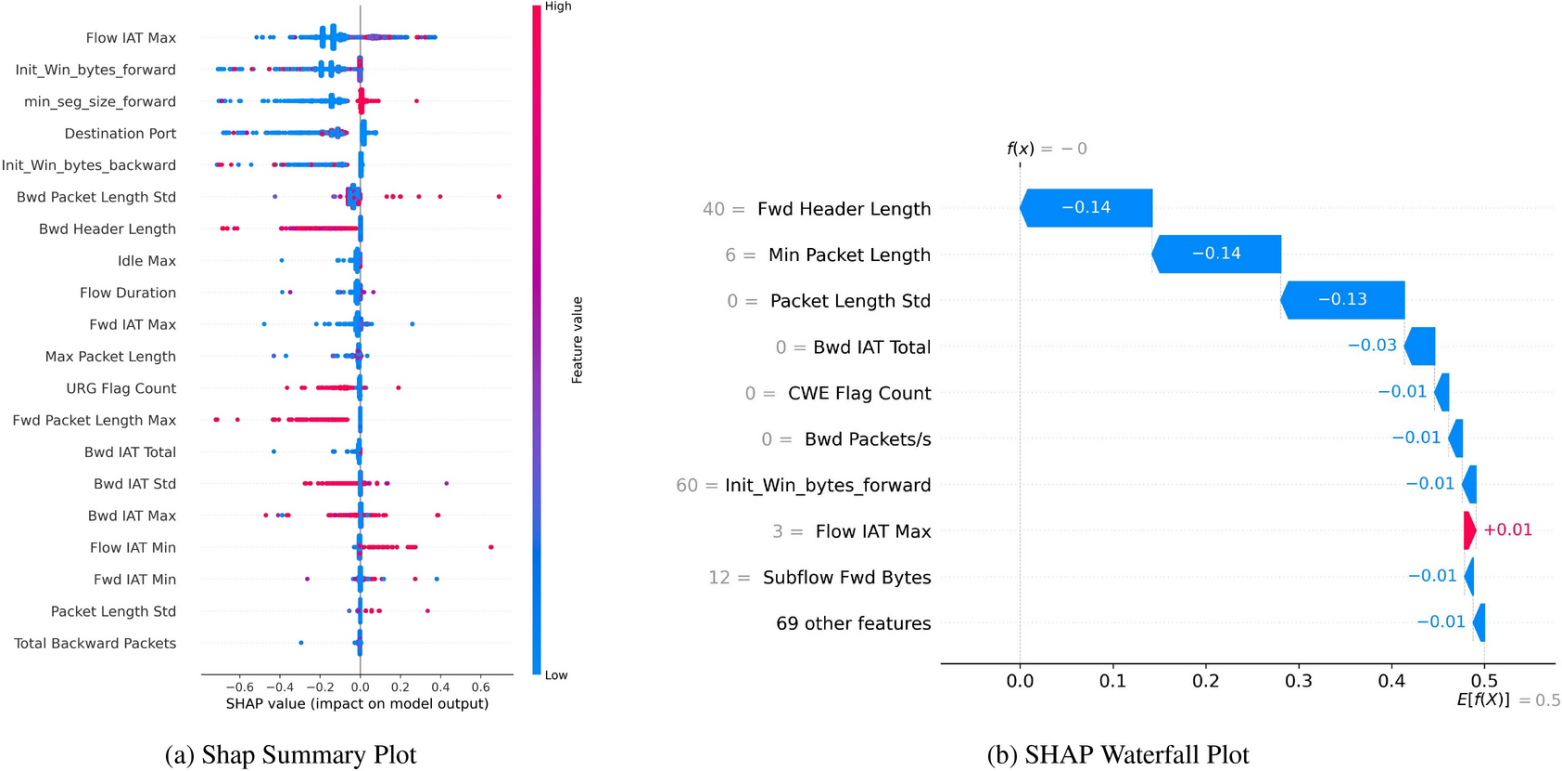
SHAP without SMOTE, indicating robust bagging and boosting binary classification performance.

Figure 17a shows features on the left side of the chart, and each feature shows the importance of a dot in the bar chart. For instance, according to this chart, “Flow IAT Max” has an absolute value as red dots, an essential feature. The range of SHAP values for this feature is about -0.6 to 0.6, So it is a significant feature for the model’s prediction. A high value of this feature (red) will increase the prediction value, but a low value (blue) will decrease the prediction. Another example of this feature is “Init Win bytes forward”. The blue spot on the left indicates a negative value, a reasonable feature to increase the impact. However, the red part suggests that if the value of “Init Win bytes forward” is too high, it impacts the model’s decision differently.

**Fig. 18:**
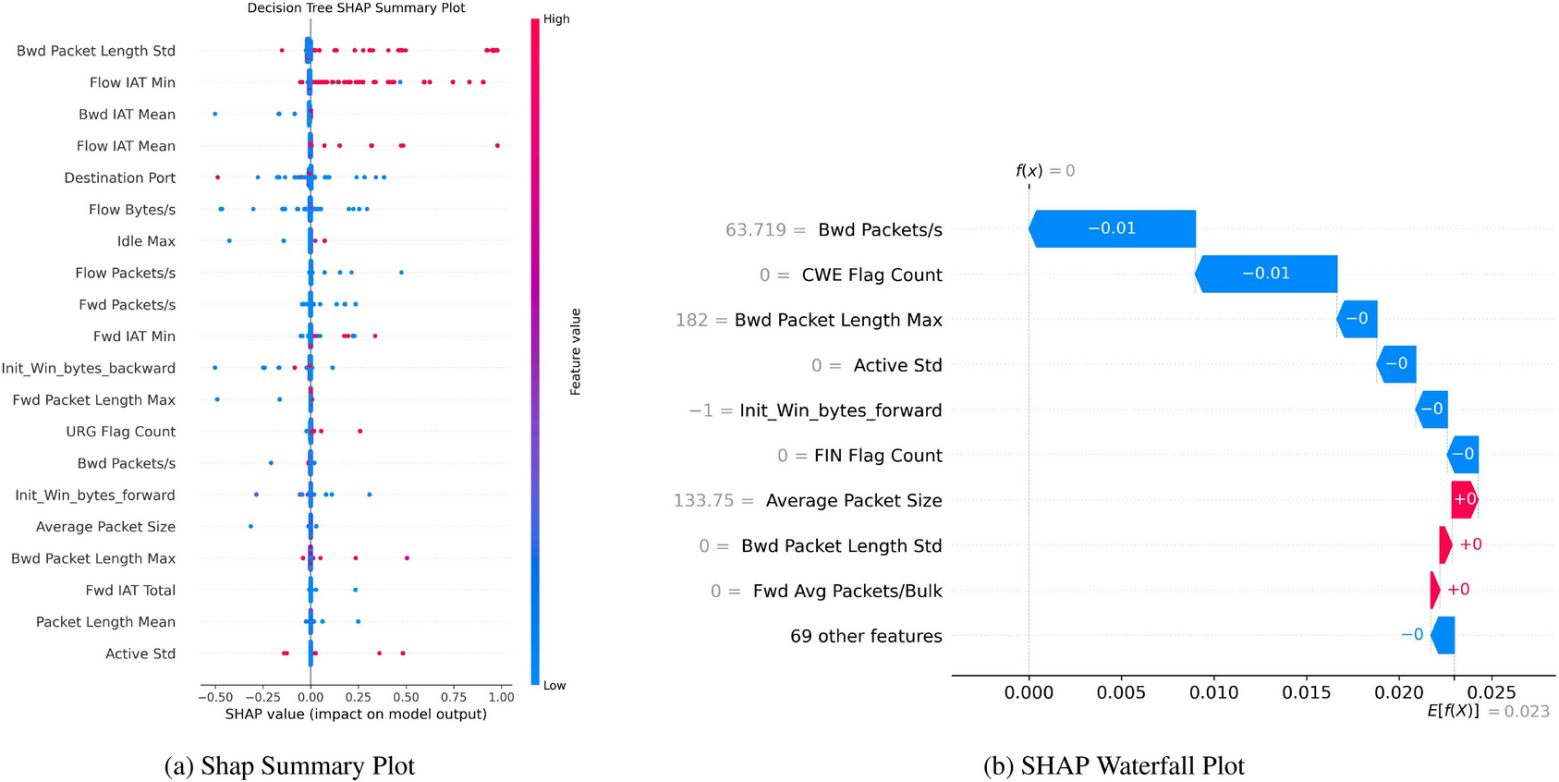
SHAP without SMOTE indicating robust boosting on residuals’ binary classification performance.

Figure 18a—SHAP Summary Plot describes a distribution of SHAP values for a particular x-axis feature, quantifying how much this feature influences the model’s predictions with positive or negative values. The spread on the chart shows extremities that have a strong influence on the predictions of either positive class or negative class, as can be observed for “Bwd Packet Length Std”, “Flow IAT Min”, and “Bwd IAT Mean”. They take positive values if they are high, reaching the highest spread. “Idle Max” and “Active Std” have a more balanced distribution around 0 (the middle) due to minor or inconclusive influence on the model, respectively.

**Fig. 19:**
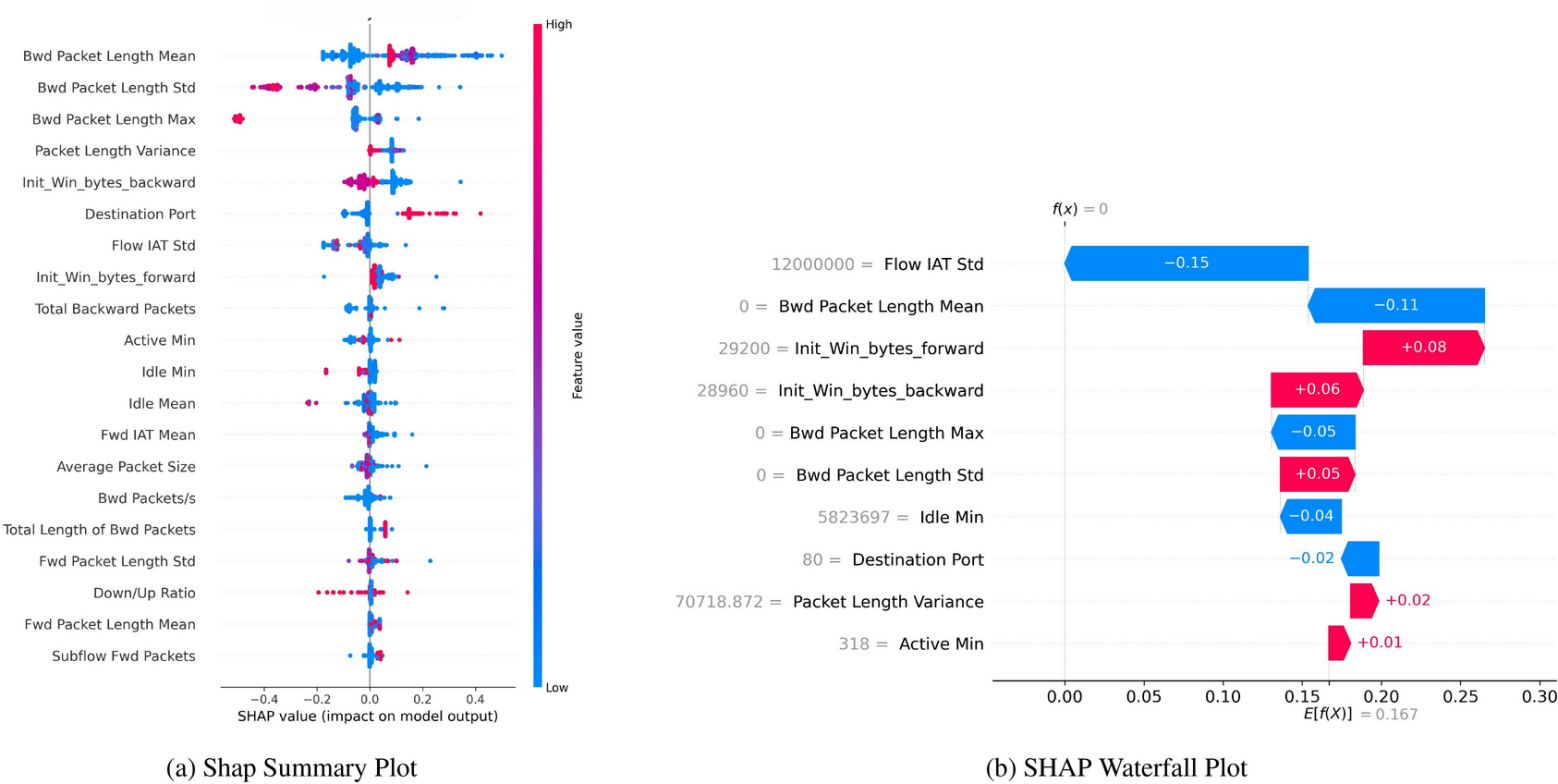
SHAP with SMOTE indicating robust bagging and boosting multiclass classification performance.

The SHAP Summary Plot Fig. 19a shows the SHAP values of each feature per sample over the entire dataset. This plot emphasizes the features’ contribution to the decision boundary prediction. The three most dominant features are ‘Bwd Packet Length Std’, ‘Bwd Packet Length Mean’, and ‘Flow IAT Std’. The dashed line at the zero value of the feature indicates the decision boundary. Below this line, the observation is scored as a planted flag, while above it is a legitimate flag. The lower the value of a particular feature, the lower the chance for a legitimate flag score by the model. The color bar represents the general distribution of the feature set. The red color corresponds to high and blue to low feature values.

**Fig. 20:**
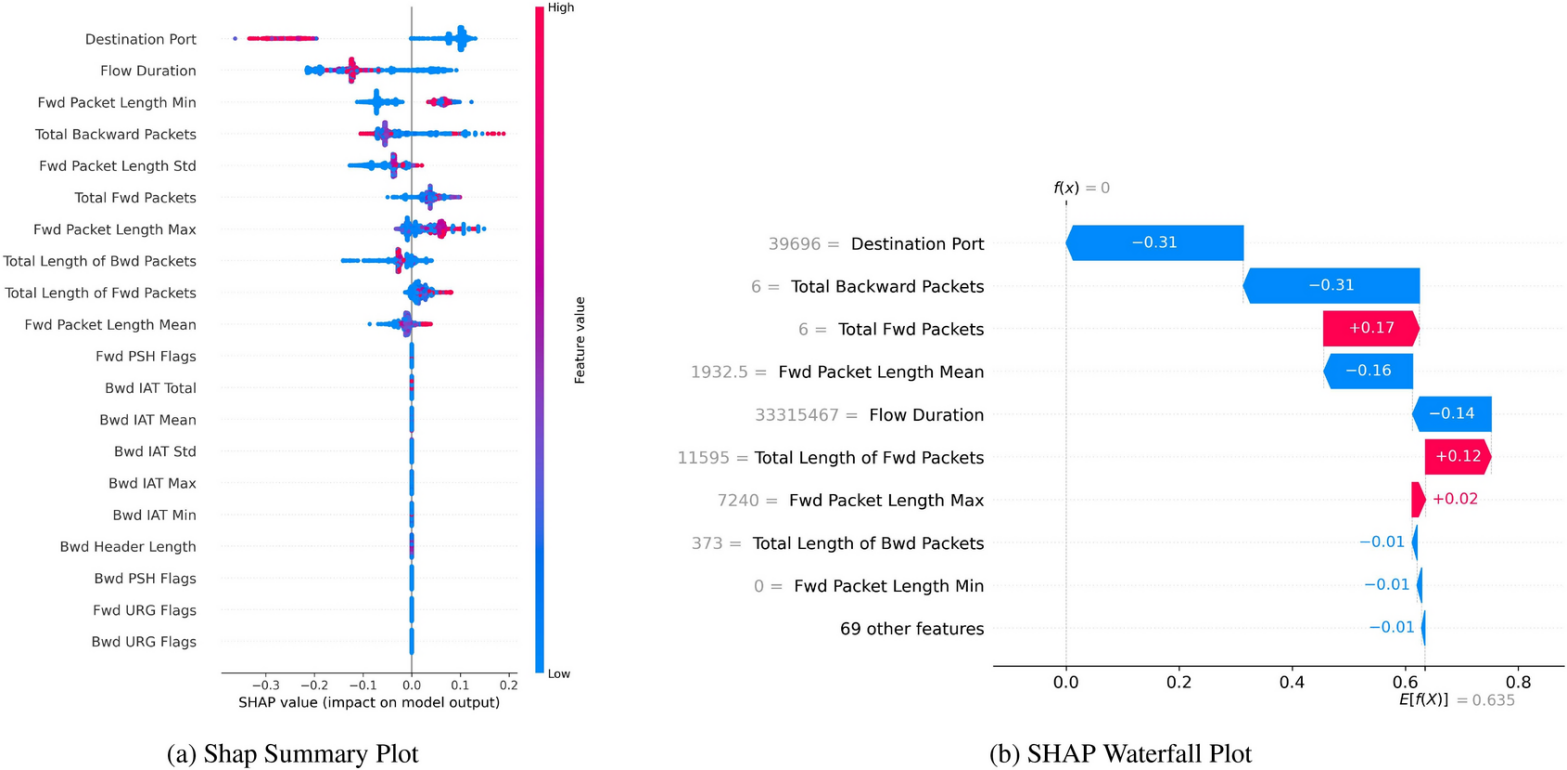
SHAP without SMOTE indicating robust Bagging and Boosting multiclass classification performance.

Figure 20a Features like ‘Bwd Packet Length Std’, ‘Idle Min’, and ‘Bwd Packet Length Max’ are the most influential as they have the highest SHAP values, an almost pure positive score in the case of ‘Bwd Packet Length Std’. The color gradient from blue to red signifies how varying feature values impact the model’s prediction, providing us with a visual understanding of how changes in feature values contribute to the final classification. High values of select features bring about considerable shifts in the model’s prediction score, demonstrating their importance in classification.

**Fig. 21:**
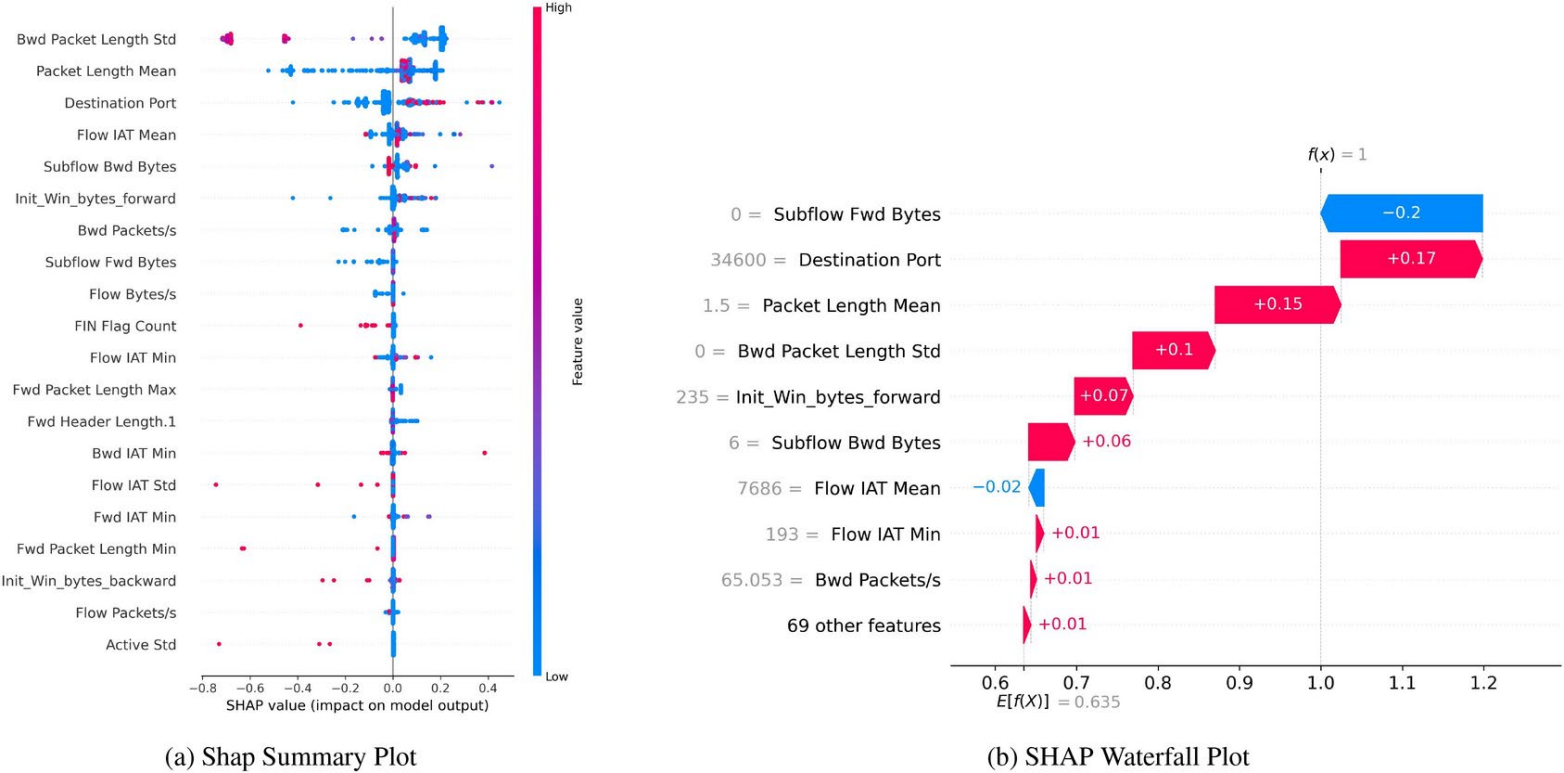
The figure shows the SHAP Summary Plot, Shap Waterfall Plot. This visualization provides an overview of the impact of features on model prediction without SMOTE, indicating robust Boosting on Residuals multiclass classification performance.

SHAP Summary Plot Fig. 21a depicts the distribution of SHAP values for each feature concerning the model’s output. The visualization on the left shows three features, the first being ’ActiveMin’, ’Bwd Packet Length Std’, and the ’Bwd Packet Length Mean’, which holds strong correlations in influencing the model’s predicted output. Specifically on the ’Active Min’ feature, the SHAP values range from -0.2 to +0.4. A color gradient from red represents the feature values, indicating a high value minimized by the model, to blue, which is the low value.

**Fig. 22:**
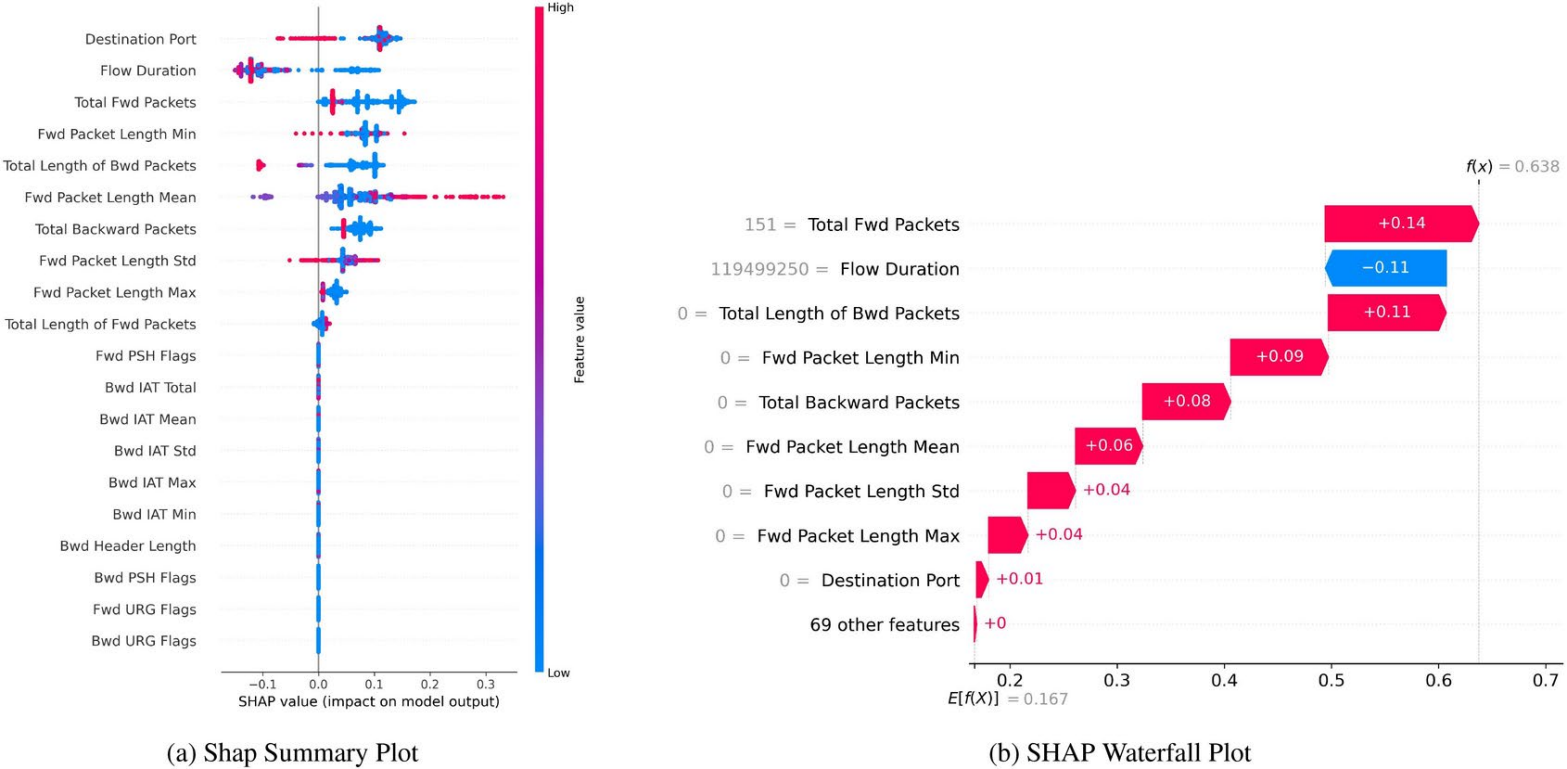
SHAP with SMOTE indicating robust boosting on residuals’ multiclass classification performance.

Figure 22a explains the high variance of features like ‘Destination Port’, ‘Flow Duration’, and ‘Total Fwd Packets’ as well as the low variance of features like ‘Bwd IAT Mean’ and ‘Bwd IAT Std’ indicates that the former group is more important for the classification task than the latter, which are less critical, and the classifier learns a joint interaction of the six features represented by salient positive feature importance.

#### SHAP waterfall plot

The Waterfall Plot Fig. 15b depicts these feature impacts cumulatively for a single instance: starting from an initial prediction and then adding in, or subtracting, the SHAP value of the feature with the most significant impact on the prediction, and so forth until the final prediction is attained. For example, “Fwd Packet Length Std” and “Flow IAT Max” have a significant positive impact, and “Idle Std” has a negative effect.

The Waterfall Plot Fig. 16b lists feature impacts in all cumulative increments for the given instance, thus revealing the evolution in each FWD score step by step. For example, the binary feature “Flow IAT Max” reduces the prediction by 0.25, “Packet Length Variance” and “Init Win bytes forward” reduce the prediction by 0.25 and 0.11, respectively, at the given step; meanwhile, the features “URG Flag Count” and “ACK Flag Count” increase the prediction by 0.09 and 0.05 respectively.

SHAP Waterfall Plot Fig. 17b illustrates the importance of features. Features, such as “min seg size forward”, negatively impact the model’s decision with high values and a positive impact with low values.

The Waterfall Plot Fig. 17b offers the cumulative (from most positive to most negative) perspective on feature contribution to a specific prediction. It starts with a certain baseline and then informs how the single feature shuttered the final prediction value. In the typical case, “Fwd Header Length” discloses how much one’s value action can effectively shrink or boost the prediction by decreasing it by 0.14 “Min Packet Length” and “Packet Length Std” then show how much is possible on top of the base (0.14 and 0.13 respectively). At the same time, “Flow IAT Max” visualizes a small positive effect, increasing it by 0.01. Such visualization lets us understand how each feature pushes the model’s prediction from the base value to the target.

Figure 18 - It shows how specific values of the features contribute to each single prediction: The SHAP Waterfall Plot Figure 18(b) starts with the base value (E[f(x)]) of 0.023, which of itself represents the average model output. After that, it is possible to observe how single features reduce or increase that base value to obtain the final prediction. For instance, ‘Bwd Packets’ and ‘CWE Flag Count’ reduce them by 0.01 each, while ‘Fwd Avg Packets Bulk’ adds 0.01. Other features - called ’Bwd Packet Length Max’ and ’Init Win bytes forward’ - are responsible for more minor variations. Visualization of the interaction between multiple features to calculate the final output of the model.

The SHAP Waterfall Plot Fig. 19b below depicts the contribution of individual features to the prediction. ‘Flow IAT Std’ features a considerable downward effect (-0.15), whereas ‘Init Win bytes forward’ and ‘Init Win bytes backward’ positively impact the prediction (+0.08 and +0.06, respectively). It elucidates how each feature pushes and pulls the prediction up or down from the input. Such visual representations make it easier to comprehend the predictor’s decision-making process.

Figure 22b is a SHAP Waterfall Plot. It is important to note that for this particular case, ’Total Fwd Packets’ and ’Flow Duration’ have the highest absolute SHAP values. +0.14 and -0.11 are the highest contributing variables to increase and decrease the prediction, respectively. At the same time, ’Fwd Packet Length Min’ and ’Total Length of Bwd Packets’, two other variables, also contribute positively to increasing the prediction.

**Fig. 23:**
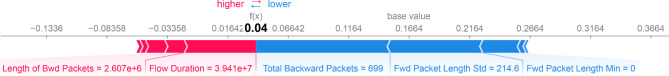
SHAP with SMOTE indicating robust bagging and boosting multiclass classification performance.

The SHAP waterfall plot in Fig. 20b details the impact of selected features on a specific prediction where the predicted class is positive (f(x) = 1). Each bar indicates how each feature contributes to the prediction score, whether adding to the final prediction or subtracting from it. The features “Subflow Bwd Bytes” and “Destination Port” contribute very positively, while “Fwd Packet Length Max” contributes very negatively. In comparison, Fig. 23b shows that ‘Subflow Bwd Bytes’ with a SHAP value of +0.14 pulls the prediction towards class 1, and ‘Flow IAT Mean’ with +0.09 is another contributing feature with a positive value. ‘Fwd Packet Length Max’ with -0.14 and ‘Flow Bytes/s’ -0.02 pull the outcome towards class 0. This breakdown provides crucial insight into the impact of a particular feature on the final prediction.

For instance, SHAP Waterfall Plot Fig. 21b describes the reasoning behind the class 1 prediction is that ’Subflow Fwd Bytes’ contributes +0.20 while ‘Packet Length Mean’ contributes +0.15, on the other hand ‘Flow IAT Mean’ contributes -0.02 and it pulls the prediction towards class 0. Importantly, this series of observations demonstrates the additive and cumulative effect of features on the model’s decision.

#### SHAP force plot

**Fig. 24:**
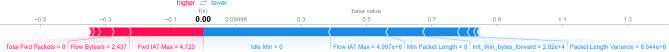
SHAP with SMOTE indicating robust bagging and boosting binary classification performance.

Figure 24 depicts a SHAP Force Plot of how specific features influence a single prediction. The base value for each feature, such as ’Total Fwd Packets’ and ’Fwd IAT Max’, is moved higher or lower by the feature in question. This plot exemplifies the interactions between features and the cumulative effect of all features on the final prediction.

**Fig. 25:**
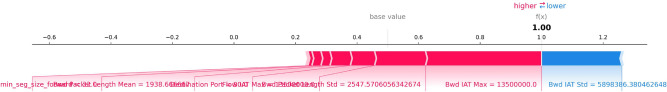
SHAP with SMOTE, indicating robust boosting of residuals’ binary classification performance.

Figure 25 shows how the feature values contribute to a prediction. For the prediction shown earlier, values for “min seg size forward”, “Packet Length Mean” and “Destination Port” have lifted the base value above the predicted value. For instance, a value for “Bwd IAT Max” is responsible for the highest increase and “Bwd IAT Std” for the slightest but positive lift. The combined nature of the increase/decrease is seen through the alternating pattern of these two features, with high and low feature values.

**Fig. 26:**
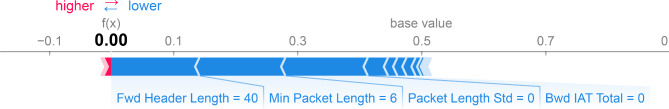
SHAP without SMOTE indicating robust bagging and boosting binary classification performance.

Figure 26 shows how specific features contribute to a single prediction. Starting from a baseline value, features like ’Fwd Header Length’ will further plummet the prediction, while ’Min Packet Length’ pushes it lower. The combo of ’Packet Length Std’ and ’Bwd IAT Total’ contributes minimally to the effect, but one feature has a very positive impact on the prediction: ’Flow IAT Max’. This plot shows the additive effect of interacting with these features, leading us to the final prediction.

**Fig. 27:**
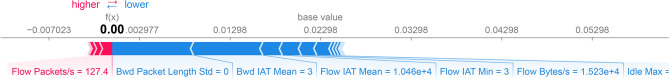
SHAP without SMOTE indicating robust boosting on residuals’ binary classification performance.

Figure 27 illustrates that each feature adds to propel the prediction up or down from the default score. Notably, the feature that drops the prediction the most relative to the baseline is ’Flow Packets/s = 127.4372372’. In contrast, features such as ’Bwd Packet Length Std = 0.0’, ’Bwd IAT Mean = 3.0’, ’Flow IAT Mean = 10462.66667’, and ’Flow IAT Min = 3.0’ push up the prediction. The diagnosis is precise: Boosting on Residuals performs robustly on binary classification.

In Fig. 23, features such as ‘Fwd Packet Length Max’ (maximum forward packet length), ‘Flow Duration’, ‘RSSI’, and ‘Fwd Short Packets’ have a negative influence, driving the prediction towards zero. On the other hand, ‘Total Backward Packets’ and ‘Fwd Packet Length Std’ (standard deviation of forward packet length) positively influence the prediction upwards, though to a lesser extent. The contributions are quantified, demonstrating how each feature impacts the value of the model prediction by a certain amount. The model finally arrives at an output of prediction value 0.04. Figure 23 plays a pivotal role in understanding how features contribute to the change of output value, and, ultimately, the decision model provides insights into feature interactions in network traffic classification.

**Fig. 28:**
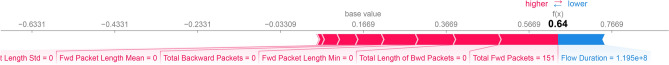
SHAP with SMOTE indicating robust boosting on residuals’ multiclass classification performance.

Figure 28 illustrates the cumulative effect of the features moving the prediction from the baseline to the final value of 0.64. Features like ‘Flow Duration’ negatively impact the prediction lower, while features such as ‘Fwd Packet Length Max’ and ‘Total Length of Bwd Packets’ push the prediction higher.

**Fig. 29:**
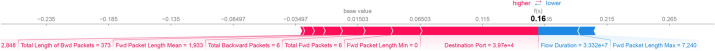
SHAP without SMOTE indicating robust Bagging and Boosting multiclass classification performance.

As seen in Fig. 29, the SHAP Force Plot decomposes the prediction of each feature and indicates each feature’s relative importance, linear significance, and directional effect. The ‘Destination Port’ is a crucial feature that strongly pulls the prediction to the higher side. However, ‘flow duration’ strongly affects the prediction’s return negatively. Other features like ‘pack lengths’ and ‘counts’ bring more specificity (how certain) around the base value, leading to the final output of 0.16 for the classification.

**Fig. 30:**
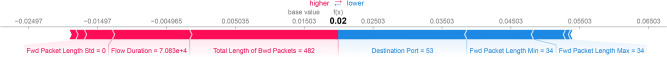
The figure shows the SHAP Force Plot. This visualization provides an overview of the impact of features on model prediction without SMOTE, indicating robust Boosting on Residuals multiclass classification performance.

Figure 30 shows how much the individual features contribute to the model’s responses in terms of magnitude and direction. The features ‘Fwd Packet Length Std’ and ‘Flow Duration’ on the left side of the plot have a red colored marker and contribute negatively to the prediction. The feature ‘Fwd Packet Length Std’, with a value of 0.0, pushes the prediction to the left. In contrast, the feature ‘Flow Duration’, with a value of 70832.0, pushes the model’s prediction of less than 50 Max Value to the left, decreasing the model’s output.

Then, on the right, features like ‘Destination Port’ and ‘Fwd Packet Length Min’ are shown in blue because they again increase the prediction - but this time, it’s a positive effect. Those features have values of 53.0 and 34.0, pushing the model output toward the right. The vertical grey line is the median model output, and the mean values for the specific prediction show up.

### LIME analysis

The LIME analysis shows a model predictive probability for classifying between “normal” and “attack” classes in binary and multiclass classification tasks with and without SMOTE to handle class imbalance. Features calculated to increase the predicted probability of an average (class 0) sample are indicated in blue. In contrast, features calculated to increase the expected probability of an attack (class 1) sample are displayed in orange.

**Fig. 31:**
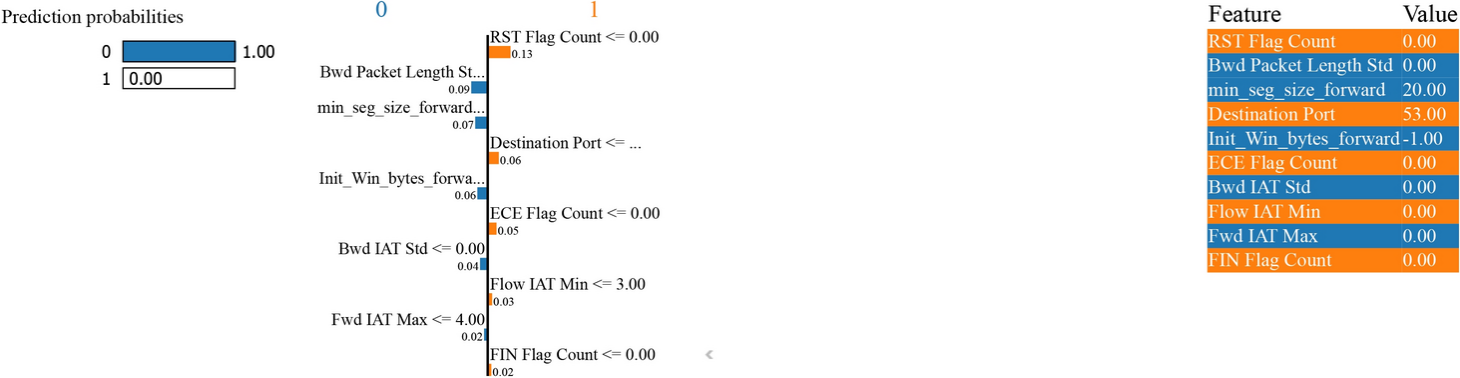
LIME for bagging and boosting binary classification performance with SMOTE.

Figure 31 - For instance, ‘Bwd Packet Length Std’ with a value of 0.0 and ‘Init Win bytes forward’ with a value of -1.0 both have a negative prediction impact on the ‘attack’ class, making the decision edge in favour of ‘normal’ class as both feature’s contribution is beneficial for it. If the ‘Destination Port’ has a value of 53.0 or ‘min seg size forward’ with a value of 20.0, a positive impact is achieved on the ‘attack’ class. Still, their effect is dominated by the feature pulling the decision in ‘normal’ class.

**Fig. 32:**
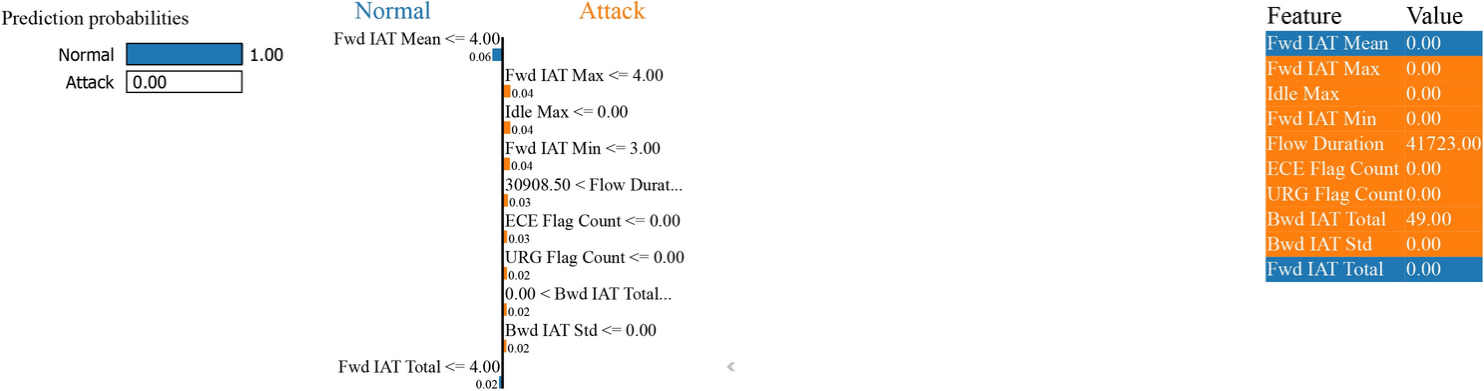
LIME for boosting on residuals’ binary classification performance with SMOTE.

Figure 32 - by classifying the “Fwd IAT Mean” feature against the threshold 4,00; if the “Fwd IAT Mean” is less than equal to 4.00, then the predicted probability is assigned “normal”, which is 1.00 for the class “normal” and 0.00 for the “attack” corresponding.

On the other hand, if “Fwd IAT Mean” is more significant than 4.00, the model begins to evaluate several different features, including “Idle Max”, “Fwd IAT Max”, “Fwd IAT Min” and “Flow Duration”, to improve upon its prediction. In that case, for instance, “Idle Max” being less than or equal to 0.00 and “Fwd IAT Max” being less than or equal to 4.00 would both assist in determining a prediction of “attack”. Meanwhile, “Fwd IAT Min” can be 3.00, “Flow Duration” can be 30908.50, and several other flag counts (FIN, ECE, ACK, and RST) can contribute variously to weighing in the decision to determine an “attack” or “normal” instance. These features along with their particular values utilized in the decision way are provided in the right side of the Figure 32: “Fwd IAT Mean”(0.00), “Idle Max” (0.00), “Fwd IAT Max” (0.00), “Fwd IAT Min” (0.00), “Flow Duration” (41723.00), “FIN Flag Count” (0.00), “ECE Flag Count”(0.00), “Fwd IAT Total” (0.00), “ACK Flag Count” (0.00), and “RST Flag Count” (0.00).

**Fig. 33:**
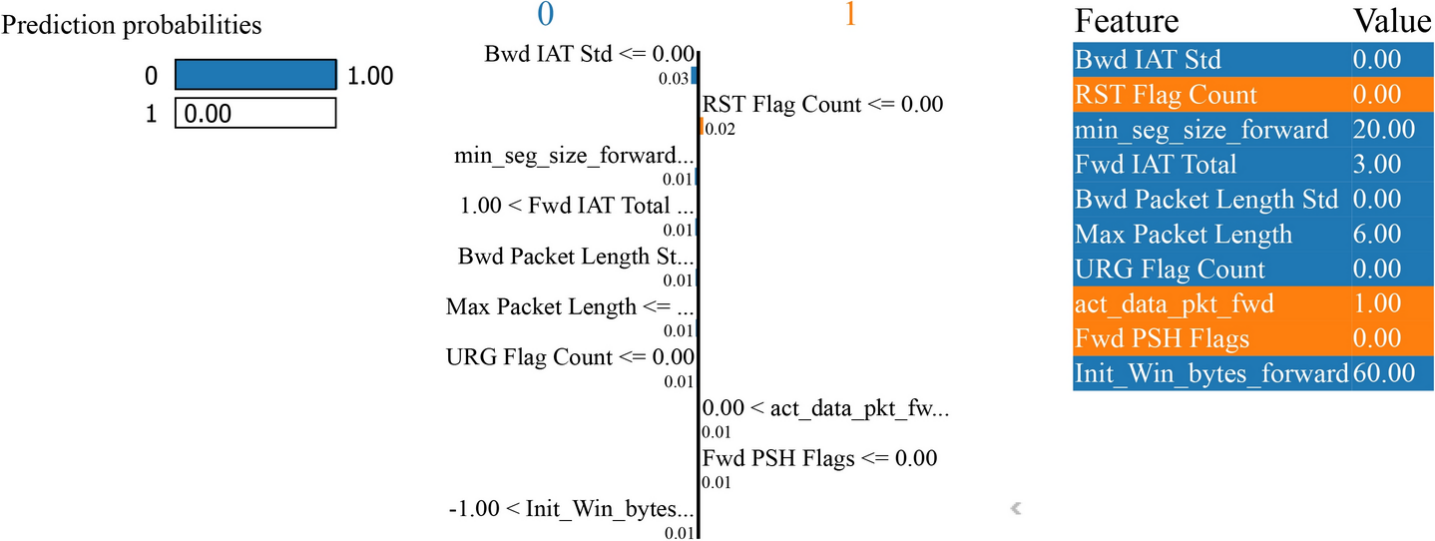
LIME for bagging and boosting binary classification performance without SMOTE.

Figure 33 - initiates the classification by evaluating the “Bwd IAT Std” feature. If “Bwd IAT Std” is less than or equal to 0.00, the model predicts a “normal” instance with a probability of 1.00 for the “normal” class and 0.00 for the “attack” class. If “Bwd IAT Std” exceeds 0.00, the model assesses additional features to refine its prediction.

The subsequent decision nodes include features like “RST Flag Count,” “Fwd IAT Total,” “min seg size forward,” and “FIN Flag Count.” For instance, if “RST Flag Count” is less than or equal to 0.00 and “Fwd IAT Total” is more significant than 1.00 but less than or equal to 3.00, the prediction pathway continues through other features such as “Init Win bytes forward” and “Max Packet Length”. The right side of the Fig. 33 lists the specific features and their values used in the decision pathway. These include “Bwd IAT Std” (0.00), “RST Flag Count” (0.00), “Fwd IAT Total” (3.00), “min seg size forward” (20.00), “FIN Flag Count” (0.00), “ECE Flag Count” (0.00), “Init Win bytes forward” (60.00), “URG Flag Count” (0.00), “Fwd IAT Min” (3.00), and “Max Packet Length” (6.00).

**Fig. 34:**
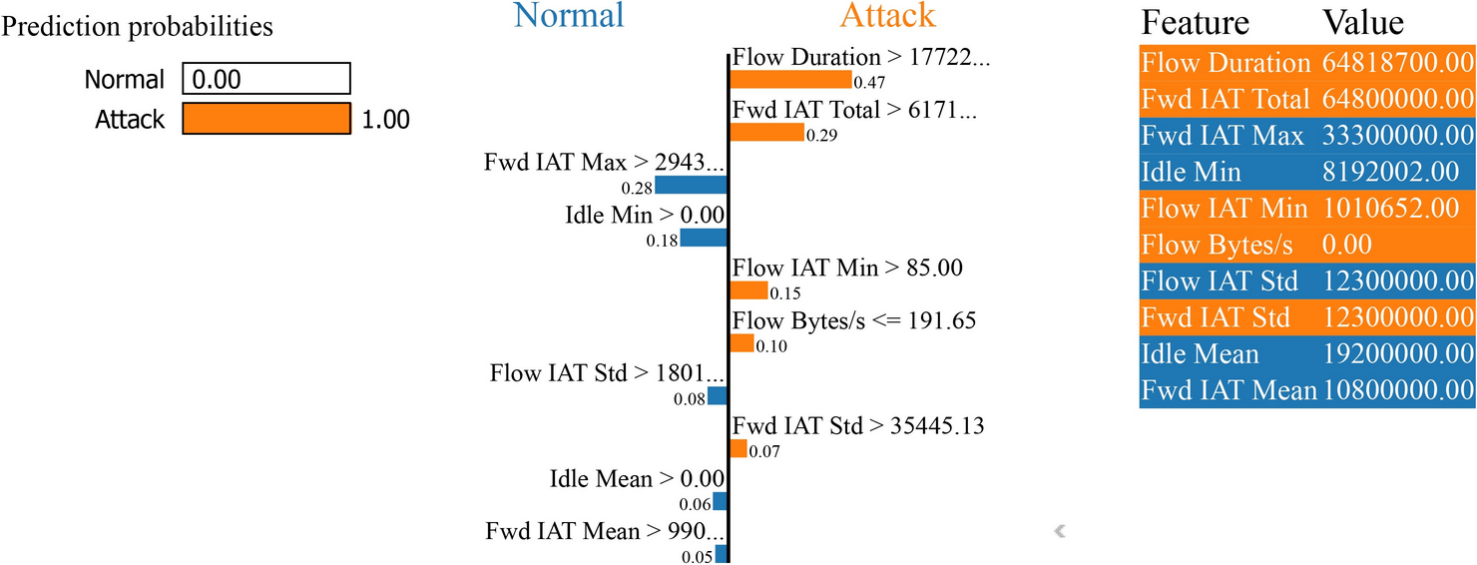
LIME for boosting on residuals’ binary classification performance without SMOTE.

Meanwhile Fig. 34 shows that key features helping the model to predict ‘attack’ are ‘Flow Duration’ with a score of 64818700.00, ‘Fwd IVT Total’ with a score of 6840000.00 and ‘Fwd IAT Std’ with a score of 35445.13 that both are orange and decide the model to predict ‘attack’. Meanwhile, features ‘Fwd IVT Max’ a with a soft core of 333000000.00 and ‘Idle Min’ a with a soft core of 819200.00 that are bluish were usually positive e to the ‘normal’ class, but they contributed less than the features that will suggest ‘attack’ arguments.

**Fig. 35:**
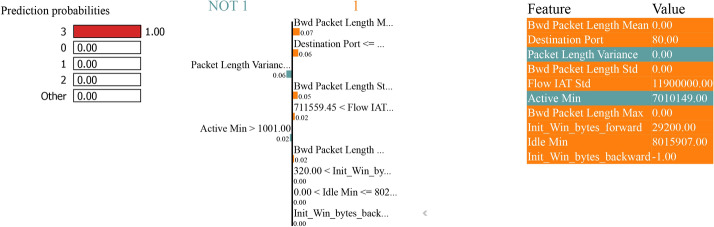
LIME for bagging and boosting multiclass classification performance with SMOTE.

The LIME plot in Fig. 35 lists the features with their corresponding values essential in the prediction. For example, the ’Bwd Packet Length Mean’ is 0.00, ’Packet Length Variance’ is 0.00, and the ’Flow IAT Std’ is 100062.00 and has high values. These values are fundamental because they make the feature selection process more transparent by explaining to a human user how each feature affects the model decision-making process. There are high values for features such as ’Active Min’ (10110048.00) and ’Idle Min’ (501097.00), meaning that the network activity has a high range and duration and can be crucial for distinguishing between different classes in a multiclass classification problem.

**Fig. 36:**
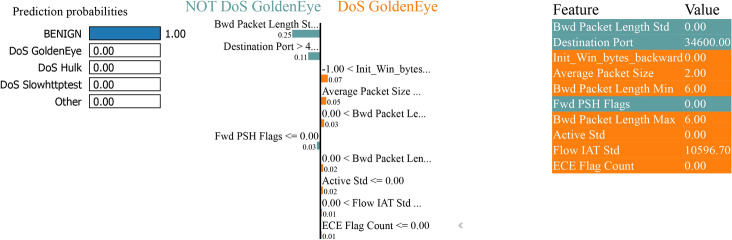
LIME for boosting on residuals’ multiclass classification performance with SMOTE.

Figure 36 shows the model predicts the instance with 100% confidence as ’BENIGN’. The middle section of the figure shows the score by each feature given the instance and how critical the features are pointing to ’BENIGN’ or ’DoS GoldenEye’. The probability of being ’BENIGN’ is very high in this case. The figure elaborates on the top features that impact the prediction by listing the feature names and the contributions. The ’Bwd Packet Length Std’ was the most contributory feature, followed by ’Destination Port’ and ’Average Packet Size’. The values depicted beside each feature name are the values that contributed to the final prediction. It could be positive, which causes bias in the final decision towards that class, or negative, which biases the final decision away from that class. This visualization shows how Boosting on Residuals with SMOTE works and how specific features such as ’Bwd Packet Length Std’ and ’Destination Port’ are critically important in predicting the model.

**Fig. 37:**
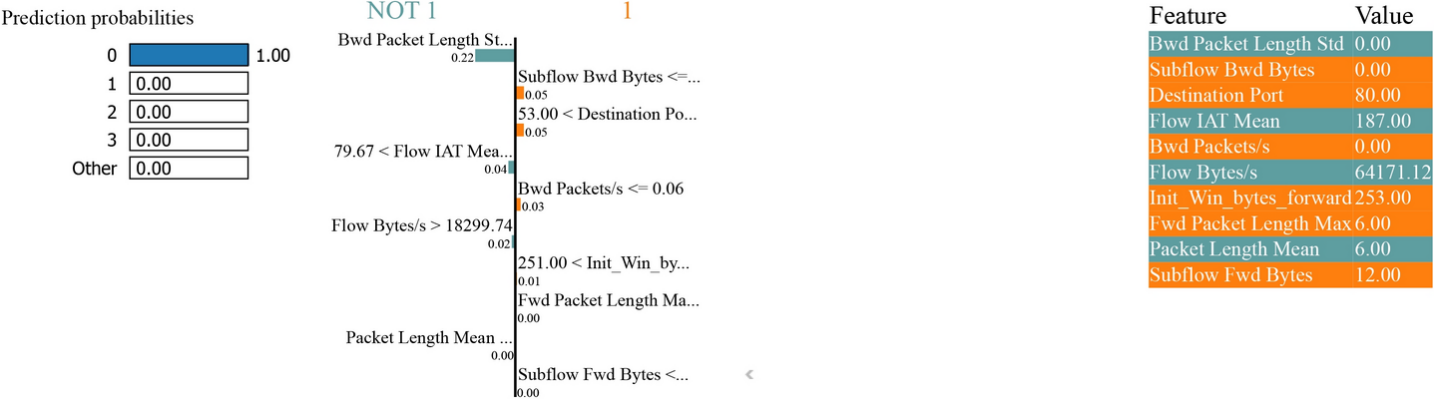
LIME for Bagging and Boosting multiclass classification performance without SMOTE.

Figure 37 also shows the prediction probabilities representing the model confidence - 100% probability the instance belongs to class’ 0’ as the instance is NOT 1. The feature,’ Bwd Packet Length Std’, strongly contributes to the prediction. This feature alone can confidently classify an instance as 0 when the value is high. Other features, such as ’Destination Port’ with a value of 53, ’Subflow Bwd Bytes’ with a value of 353, and ’Flow IAT Mean’ with a value of 1178, contribute positively. Whereas ’Bwd Packet Length Std’ shows the highest enumeration, ’Destination Port’ and ’Subflow Bwd Bytes’ have the second highest. The value of the features highlights their impact assessment, and the positive values contribute to the final prediction.

Figure 37 is a simple representation that underscores the model’s capability to learn from a limited number of critical features, such as ’Bwd Packet Length Std’ and ’Destination Port’, and perform a robust classification performance without using SMOTE.

**Fig. 38:**
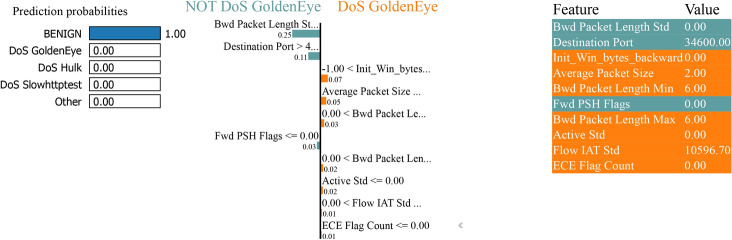
This figure shows the model’s predictive probabilities for indicating robust Boosting on residual multiclass classification performance without SMOTE.

Figure 38 illustrates the model’s predictive probabilities of robust Boosting on Residuals multiclass classification performance without SMOTE for ’DoS GoldenEye’ attacks. It can be seen that the model’s predicted outcome shows a 1.00 confidence for predicting the robust scheme in the classification network when it predicted ’BENIGN’. On the other hand, with the probability 0.00, it predicted ’DoS GoldenEye’. Several features account for the course mentioned above of events, including ’Bwd Packet Length Std’ (0.00), ’Destination Port’ (34600.00), ’Average Packet Size’ (2.00), ’Init_Win_bytes_backward’ (0.00), ’Bwd Packets’ (65.00), ’Flow IAT Min’ (49.00), ’URG Flag Count’ (0.00), ’Idle Min’ (0.00), and ’Bwd Packet Length Min’ (0.00).

In Fig. 38, the ’Bwd Packet Length Std’ has the most significant variable importance of the dataset (0.00), followed by ’Destination Port’ (34600.00) and then ’Average Packet Size’ (2.00). Considering each feature’s importance is crucial to understanding how the model is impacted by any single feature, thus enhancing the transparency of a machine learning model’s contribution to the cybersecurity results.

To summarise, the model clearly distinguishes between benign and ’DoS GoldenEye’ attacks with a predictive probability of 1.00 for the ’BENIGN’ class. According to the feature importance analysis, the model is influenced by the following three features: ’Bwd Packet Length Std’, ’Destination Port’, and ’Average Packet Size’. Given the typical opacity of machine learning models, these insights are indispensable for making machine learning more interpretable in cybersecurity and, therefore, more valuable and reliable for decision-making in cyber-attacks at the training stage of machine learning models.

### Discussion

This section discusses the proposed model’s performance results. It explains how it performed with SHAP features selection using balancing and balancing of the CIC-IDS2017 dataset.

### Performance with SMOTE

**Fig. 39:**
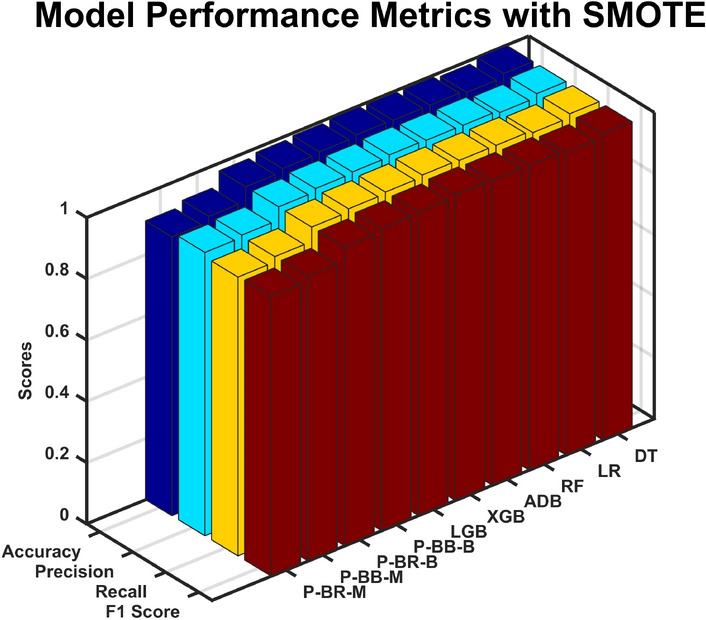
Proposed HBB-RE model performance using SMOTE.

Figure 39 presents the 3D bar chart of the intelligent state-of-the-art Bagging and Boosting model classifiers of binary and multiclass classification of the CIC-IDS2017 dataset validated by training performance with the capability of hybrid machine learning-based algorithms. Figure 39 shows a high accuracy of 98.47% and 92.75% for all measures in the intelligent model, together with binary and multiclass classification credentials. It shows its ability to learn proposed Bagging and Boosting algorithms in binary and multiclass (P-BB-B and P-BB-M) classification. However, baseline classifiers like RF, XGB & LGBM demonstrated impressive performance, with nearly 99% accuracy in both cases.

The proposed Boosting on Residual model for binary and multiclass (P-BR-B and P-BR-M) reveals positive graph slopes of near-perfect accuracy of nearly above 97.45% and over 90.99% for binary and multiclass, respectively. AdaBoost performed great in the binary and the multiclass tasks, while GDMB also performed excellently in the binary and multiclass tasks. DT, ADB RF, XGB, and LGB performed well and excellently in the hybrid binary and multiclass performance dominance for the datasets.

### Performance without SMOTE

**Fig. 40:**
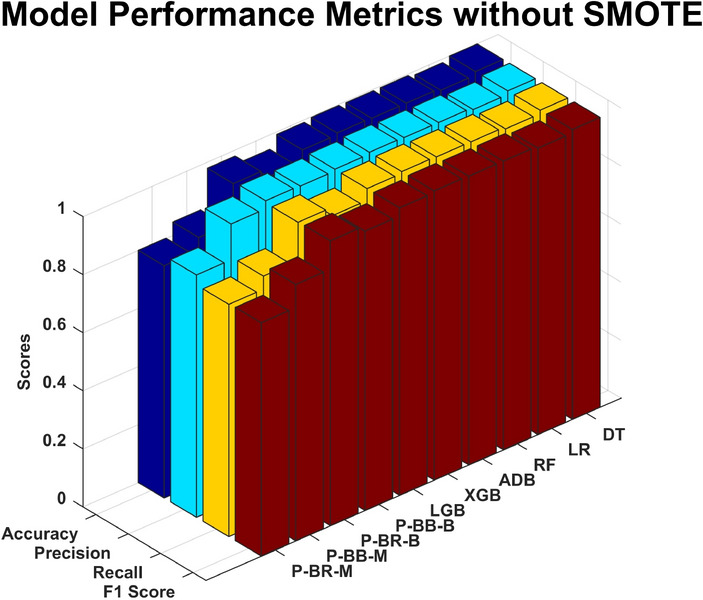
Proposed HBB-RE model performance using without SMOTE.

Figure 40 shows the near-perfect accuracy of over 94.92% and 84.52% across all measures; the state-of-the-art Bagging and Boosting model performed well in binary and multiclass (P-BB-B and P-BB-M) classification on the CIC-IDS2017 dataset. However, baseline classifiers like RF, DT, and ADB demonstrated impressive performance with nearly 99% accuracy. At the same time, other models, such as XGB and LGB, scored well in multiclass classification with SHAP features selection without SMOTE.

The Boosting on Residual model (P-BR-B and P-BR-M) dominated the binary class with 97.84% accuracy and the multiclass with over 80.01% accuracy. AdaBoost and XGB also performed well in multiclass tasks with a high accuracy of 99%. DT, RF, and ADB excelled in the hybrid dominance in binary and multiclass performance.

### Performance analysis

The performance of the proposed models demonstrates significant advancements in predictive accuracy and reliability across various configurations.

**Fig. 41:**
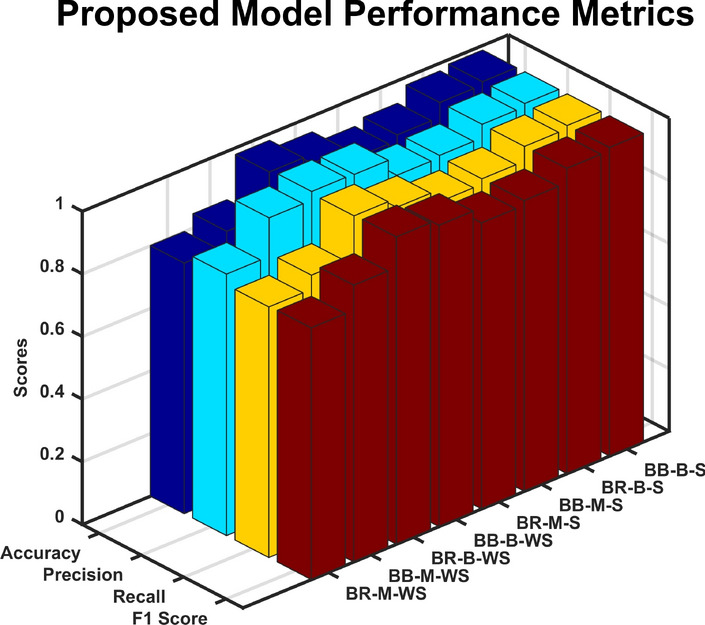
Combine HBB-RE model performance with and without SMOTE.

Figure 41 evaluates the performance of various proposed models using four key metrics: Accuracy, Precision, Recall, and F1 Score. The models include both feature selection with SMOTE (S) and without SMOTE score (WS) variants of Bagging and Boosting (BB) and Boosting on Residuals (BR), whereas (B) is for binary class and (M) is for multiclass classification. The BB-B-S model exhibits high performance, with all metrics around 98.47% to 98.48%, indicating consistent and reliable predictions. The BR-B-S model follows closely, showing slightly lower performance with scores of 97.45% across all metrics. On the other hand, the declining trend of BB-M-S drops significantly, with performances at 92.75% to 93.21%, indicating a drop in dependability of performance, and the BR-M-S has even lower performances in its metrics at 90.99% to 92.50% with another level of decline in performance.

The BB-B-WS is better according to all four metrics, scoring between 94.92% and 96.19%, with weighted scoring adding to the efficacy of the standard BB model. The BR-B-WS model has metrics of between 97.84% and 98.60%, showing the model’s robustness. The BB-M-WS model, however, although it has a range of acceptable variation, i.e., between 84.52% and 95.83%, also shows inconsistent performance. Finally, the BR-M-WS model has the lowest scores of all the proposed models, with metrics of between 80.01% and 83.49%, and the least accurate predictions on all accounts.

The data show that the weighted scoring has statistically enhanced the performance of the models and that the BR-B-WS does the best job. The modified models (BB-M-S and BR-M-S) provide good insight into the sources of performance variability and possible improvement points.

### Comparative study

This section compares the methods and feature algorithms to verify the performance of the proposed (HBB-RE) module in terms of using 100 SHAP feature selection processing. It also compares it with the state-of-the-art intrusion detection methods to demonstrate the advantages of the proposed (HBB-RE) IDS.

#### Comparison with classical methods

Classical methods such as decision trees, k-nearest neighbors (k-NN), and support vector machines (SVM) run inefficiently on high-dimensional data. They are less useful for the analysis practice. The SHAP-based method effectively identifies the relevant features and promotes model accuracy and robustness. A rich literature shows that SHAP-based methods help to interpret models and can either improve existing models or identify new approaches to improve model performance in diverse domains^44^. A general trade-off holds between model interpretability and predictive performance.

On the other hand, critical advances have led to impressive improvements in accuracy, such as neural networks and ensemble methods like random forests and gradient boosting. However, these models often have limited interpretability or cannot explain why they produce a particular output from a specific input^5^. The SHAP-based method nicely demonstrates the importance and contribution of features that should be more interpretable for non-experts.

Classical methods often implode when dealing with imbalanced datasets^45^. Combining SHAP-based feature selection with modern resampling techniques can improve performance on imbalanced datasets while maintaining interpretability. Overfitting is an obstacle in classical machine learning methods^46^. Hybrid ensemble learning techniques and SHAP-based feature selection mitigate overfitting, improve generalization, and stabilize promotion.

Moreover, classical methods generally report high accuracies but fail miserably on more meaningful measures, such as precision, recall, and F1 score^47^. Using SHAP-based feature selection, the model achieves higher accuracy and F1 scores than classical methods. The proposed method demonstrates its significance in developing robust and accurate IDPS and is the first to use SHAP-based feature selection. This type of learning is expected to aid in creating more reliable, strong, and explainable IDPS in scientific fields.

#### Comparison with state-of-the-art methods

The Table 4 illustrates various studies and proposed methods related to Feature Selection, Algorithm, Number of Features, Accuracy, and F1 Scores in Predictive Modelling. The studies are done from Study 1 to 11 in which Feature Selection methods and Algorithms differ. Feature Selection methods used are NTLEBO, HFS, RFE, Weka-ML, F1, PSO-FO-GO-GA. Algorithms are traditional ones such as Logistic Regression (LR) and Decision Tress (DT) as well as the ones that are much more advanced such as Deep Neural Networks combined with Ant Colony Optimization (DNN+ACO), LGBM, XGBoost, Light Gradient Boosting Machine, Random Forest, Artificial Neural Networks (ANN). Abbreviations used in this study include P (Proposed), B (Binary), M (Multi), S (SMOTE), and W (Without).

The number of features selected for models in the studies ranges from 5 (Study 8)^48^ to 30 (Study 11)^49^. This range suggests that feature selection and dimensionality reduction may have been performed differently in the different studies, using various ad hoc strategies to optimize model performance. The overall model performance in accuracy and analysis is given by 75.66% (Study 9)^50^ to 98.25% (Study 2)^51^. At the same time, for F1 scores - which measure a balance between precision and recall- the values range from 77.28% (Study 10)^52^ to 97% (Study 1)^53^.

**Table 4:** Comparison of Feature Selection and Algorithms.

Study	Ref	Feature Sel.	Algorithm	No. Features	Accuracy	F1 Score
Study 1	^53^	NTLBO	LR	22	97.00	97.00
Study 2	^51^	-	DNN+ACO	-	98.25	-
Study 3	^54^	HFS	LGBM	24	97.73	-
Study 4	^55^	-	RF+XGB	12	96.36	-
Study 5	^55^	-	DT+XGB	12	96.30	-
Study 6	^56^	RFE	RF	13	84.30	84.04
Study 7	^56^	RFE	DT	13	84.35	84.36
Study 8	^48^	Weka-ML	RF	5	82.99	81.40
Study 9	^50^	F1	RF	11	75.66	77.32
Study 10	^52^	XGBoost	ANN	19	77.51	77.28
Study 11	^49^	PSO-FO-GA	J48	30	90.48	90.17
P-B-S	–	SHAP	HBB-RE	100	98.47	98.47
P-B-S	–	SHAP	HBB-RE	100	97.45	97.45
P-M-S	–	SHAP	HBB-RE	100	92.75	92.81
P-M-S	–	SHAP	HBB-RE	100	90.99	91.40
P-B-W/S	–	SHAP	HBB-RE	100	94.92	96.19
P-B-W/S	–	SHAP	HBB-RE	100	97.48	97.48
P-M-W/S	–	SHAP	HBB-RE	100	84.52	88.34
P-M-W/S	–	SHAP	HBB-RE	100	80.01	80.34

These state-of-the-art studies are compared with the proposed methods - Proposed-B-SMOTE, Proposed-M-SMOTE, and Proposed-B-W/SMOTE - which use SHAP for feature selection and HBB-RE as the algorithm, all of which use 100 features. Using SMOTE as a baseline technique, the accuracy for Proposed-B-SMOTE is 98.47%, and the F1 is 98.47%, showing a very high predictive performance. As for Proposed-M-SMOTE, the accuracy is 84.52% to 92.75%, and the F1 scores are 84.34% to 92.81%, showing that the value varies based on the modification. As for Proposed-B-W/SMOTE, the accuracy is 94.92%, and the F1 is 96.19%, which also shows a strong performance effect.

The proposed methods outperform state-of-the-art algorithms in accuracy and F1 score. Table 4 shows that a Proposed-B-W/SMOTE has the highest accuracy of 98.47%, slightly more than the most significant accuracy, 98.25%, in (Study 2). Similarly, F1 scores are also higher than those of the existing studies, which is 96.19%. Compared to the state-of-the-art methods, achieving a higher F1 score using the proposed methods is higher than most studies. The proposed methods have improved overall accuracy, especially with standardizing to 100 features and using SHAP for feature selection. It makes the methods better compared to the state-of-the-art methods. The proposed methods use advanced feature selection techniques combined with resampling techniques to improve the performance of ML models compared to many state-of-the-art methods in improving model accuracy and balancing between precision and recall.

## Conclusion and future direction

The SHAP-based feature selection, a hybrid form of bagging and boosting algorithms used for detection, and the residual correction are huge advancements towards better-designed, highly supple, and exceptionally robust-accuracy IDPSs. These statistical approaches mutually enhanced the predictive and explanatory models of intrusion detection. Simultaneously, the combined statistical approach resolves two modeling pitfalls in traditional cyber-security approaches. With the stacking approach, model boosting resolves concerns such as overfitting the data, and model averaging resolves the inherent credibility crisis faced by the intrusion detection models in the ex-ante environment. This solution can open a future research avenue to test other statistical innovations, if possible, to boost the predictive power of IDPSs.

Precise areas on which the researchers can build up their findings include the following: Firstly, identifying how these hybrid methods adapt to the dynamic nature of evolving cyber threats is of great importance. Another critical area could be how real-time data processing impacts the effectiveness and efficiency of IDPS models, showing their practical usability. The scalability of these models in large-scale network environments and their resilience against advanced persistent threats are other aspects to be considered. Finally, this trade-off between model complexity and interpretability will contribute to designing more transparent and user-friendly intrusion detection systems. Answering such questions and focusing on the highlighted areas would lead to further steps in improving this field by offering robust and adaptive cybersecurity solutions.

## Data Availability

The dataset used in this study is publicly available at https://www.unb.ca/cic/datasets/ids-2017.html.
